# Adding Active Slot Joint Larger Broadcast Radius for Fast Code Dissemination in WSNs

**DOI:** 10.3390/s18114055

**Published:** 2018-11-20

**Authors:** Wei Yang, Wei Liu, Zhiwen Zeng, Anfeng Liu, Guosheng Huang, Neal N. Xiong, Zhiping Cai

**Affiliations:** 1 School of Information Science and Engineering, Central South University, Changsha 410083, China; xlh950807@csu.edu.cn (W.Y.); zengzhiwen@mail.csu.edu.cn (Z.Z.); 2 School of Informatics, Hunan University of Chinese Medicine, Changsha 410208, China; weiliu@csu.edu.cn; 3 The State Key Laboratory of Industrial Control Technology, Zhejiang University, Hangzhou 310027, China; 4 School of Information Science and Engineering, Hunan First Normal University, Changsha 410205, China; hgsheng@mail.sysu.edu.cn; 5 Department of Mathematics and Computer Science, Northeastern State University, Tahlequah, OK 74464, USA; xiongnaixue@gmail.com; 6 Department of Network Engineering, School of Computer, National University of Defense Technology, Changsha 410073, China; zpcai@nudt.edu.cn

**Keywords:** wireless sensor networks, adding active slot, energy efficiency, codes dissemination, minimum-transmission broadcast, delay

## Abstract

By using Software Defined Network (SDN) technology, senor nodes can get updated program code which can provide new features, so it has received extensive attention. How to effectively spread code to each node fast is a challenge issue in wireless sensor networks (WSNs). In this paper, an Adding Active Slot joint Larger Broadcast Radius (AAS-LBR) scheme is proposed for fast code dissemination. The AAS-LBR scheme combines the energy of data collection and code dissemination, making full use of the remaining energy in the far-sink area to increase the active slot and the broadcast radius to speed up the code dissemination. The main contributions of the proposed AAS-LBR scheme are the following: (1) Make full use of the remaining energy of the far sink area to expand the broadcast radius, so that the node broadcasts a longer distance. The wide range of broadcasts makes the number of nodes receiving code more, which speeds up the spread of code dissemination. (2) AAS-LBR uses two improved methods to further reduce the number of broadcasts and speed up the code dissemination: (a) When constructing the broadcast backbone whose nodes dominate all nodes in network and are responsible for broadcasting code, the active slot is added to the next hop node in a pipeline style on the diffusion path, which enables the code dissemination process to continue without pause. Thus, the code can quickly spread to the entire broadcast backbone. (b) For the nodes in the non-broadcast backbone whose nodes are dominated by the broadcast backbone and only for receiving code, an active slot is added coincident with its broadcast backbone’ active slot, which can reduce the time required for code dissemination and reduce the number of broadcasts. A lot of performance analysis and simulation results show that compared to previous schemed, the AAS-LBR scheme can balance energy consumption, the transmission delay can be reduced 43.09–78.69%, the number of broadcasts can be reduced 44.51–86.18% and the energy efficiency is improved by about 24.5%.

## 1. Introduction

Wireless sensor networks (WSNs) are an important component of the Internet of Things (IoTs), which have received wide attention from industry and academia [1,2,3,4]. With the development of microprocessor technology, the size of sensor nodes has become smaller and smaller, and their functions more and more powerful [5,6,7]. Sensor nodes consisting of microprocessors, storage devices, batteries, communication devices, and sensing devices are becoming promising platforms and are used in many applications such as smart cities [6], traffic information systems [7], industrial automation [8,9], public facilities and security monitoring, environmental monitoring [10,11,12,13], human health surveillance [14], wildlife protection and military applications [15,16,17,18]. Because the sensor nodes are simple in structure and easy to deploy, they can perform long-term unattended monitoring of the monitoring target, and can send the monitoring data back to the control center through the self-organizing network in time for data analysis. This can m for example, monitor and protect wild animals and control the automatic control of industrial control systems, so its research value is huge [19,20,21].

The emergence of Software Defined Network (SDN) technology [5,7,8,14,17,18] has made WSNs more powerful. SDN technology is a technology that uses re-programming technology [17,18] to replace the soft code in hardware devices. After the system or application software of the hardware system is recompiled, the hardware will have new functions and expand its application field. SDN technology is not a new concept [17,18], but it has demonstrated its unique advantages in WSNs in particular [17,18]. Since sensor nodes are generally deployed in areas that are difficult for human to reach, it is difficult to replace and upgrade them [22,23,24,25]. However, SDN technology can give full play to its advantages. Using SDN technology combined with wireless technology, the latest code can be transmitted to the sink through the remote network, and the latest code is propagated from the sink to each node in the network through wireless broadcasting. Nodes can then recompile this new program code to enable nodes to be upgraded, without having to replace hardware devices, and has great advantages in real-time, cost, and operability [5,7,8,14,17,18]. However, code dissemination in WSNs is also a challenge issue. There are mainly several challenges:

(1) How to quickly spread the code to each node in the network. Dissemination delay is the difference between the time when the sink starts the code dissemination and the time when the last node in the network receives the code. Reducing the dissemination delay is of great significance for the application of WSNs [6,7,8,12,14,17]. In general, the purpose of code dissemination is to update WSNs by adding new features, sensing new type of data, new data processing functions, and extending the field of their applications. Therefore, in the process of code dissemination, the data and data processing results of the node that first received the code are inconsistent with the nodes of the non-updated code. Obviously, this inconsistency will exist in any code updating process, however, the duration of such inconsistency is expected to be as short as possible, as this inconsistency may cause confusion and reduce the reliability of the application. For example: in large-scale crop monitoring, it is necessary to process the perceived data in near real-time. If updating the code takes a long time, it may cause data inconsistency, which will affect the correct decisions. In industrial monitoring applications where real-time requirements are very strict, the requirements are even more stringent, and the inconsistency of data is required to be as short as possible. Otherwise, it may cause significant production losses [23]. However, to quickly spread the new code to an entire network it is not an easy task. In WSNs, because the nodes are powered by battery energy, due to manufacturing costs and economic considerations, nodes should be as simple as possible and low in cost. Therefore, the capacity of the batteries is limited [26,27,28,29]. As nodes are generally deployed in places that are dangerous or difficult for humans to reach, it is generally difficult to replace the drained sensors. Therefore, how to save energy is one of the most important and hot topics of WSNs [30,31]. One of the most common methods used to save energy is the periodic sleep/awake rotation of the nodes [7,15,17] because when the node is in the sleep state, its energy consumption is less than 1/100 of its energy consumption in the awake state [32,33,34,35]. Therefore, the node should be in the sleep state as much as possible to save energy [7,15,17]. Although duty cycle-based WSNs can save energy, it causes greater delays in code dissemination [7,15,17]. Because in duty cycle-based WSNs, the working period T of the node is divided into n equal slots, and the node is only active in one of the slots which is called active slot, and in other slots sleep. When the node is in the sleep state, the nodes cannot communicate with each other. When the sender sends the code to the receiver, the sender must wait for the receiver to awake before the code can be spread. The average expected delay in this case is approximately (n−1/2) slots. The time for multi-hop code dissemination increases linearly as the number of hops increases. Thus, how to quickly spread code to the network is a challenge issue [7,15,17].
(2) How to reduce the number of broadcasts. Since the energy in wireless sensor networks is the most valuable resource, reducing the number of broadcasts can reduce the energy consumption of the network, and reducing the number of broadcasts can also shorten the time required for code to spread. Therefore, reducing the number of broadcasts in the code dissemination process is another important research issue. This problem is summarized as the minimum-transmission broadcast (MTB) problem [7,12]. In non-duty cycle WSNs, the parent node only needs to broadcast once to get all the son nodes to receive the code. In the duty cycle-based WSNs, since the slots of the son nodes awake times may be different, the parent node may need to broadcast once for each active slot of its son nodes, so the number of broadcasts is greatly increased. This shows that the MTB problem is a challenging issue in duty cycle-based WSNs.
(3) Improve network life. Lifetime has always been an important issue in WSN research. In general, reducing the energy consumption of node can extend the life of nodes. Therefore, the main way to improve the network lifetime is to reduce the energy consumption of nodes. The energy consumption of nodes is mainly due to data transmission, data reception, energy consumption during idle waiting tasks, therefore, reducing the number of broadcasts reduces the energy consumption of data operations (sending and receiving). The idle energy consumption refers to the energy consumption of the listening channel when the node is in the active state [36,37,38,39]. The key way to reduce the idle waiting energy consumption is to make the node sleep as much as possible when there is no data operation, so that the active time is as small as possible, but this will bring more delay. In other words, lifetime is closely related to the performance of delay and broadcast times, and comprehensive optimization is needed to achieve better results [40,41,42,43]. In addition, network lifetime and node lifetime in WSNs are not the same concept [44,45]. Due to the imbalance of energy consumption, in general, when data is collected by WSNs, the near-sink area node bears a large amount of data and consumes a large amount of energy, which may cause nodes in the near-sink area to die prematurely, resulting in network death [46,47,48]. According to studies [44,46], there may still be up to 90% energy remaining in the network, so the most important thing to improve the network lifetime is to improve the life of the nodes whose energy consumption is the largest. However, saving energy for the node with superfluous energy does not increase the network lifetime [47,48].

There have been some research on code dissemination. These studies mainly concern three important performance indicators of reducing code dissemination delay, the number of broadcast and network lifetime. Although these studies have achieved certain results, there are still some work worthy of further study. In summary, the main innovations of this paper are as follows:

(1) An Adding Active Slot joint Larger Broadcast Radius (AAS-LBR) scheme is proposed for reducing the number of broadcasts and fast code dissemination while retaining higher network lifetime for WSNs. The important innovation of the AAS-LBR scheme is that it can significantly improve the performance of code dissemination through increasing the active slots and increasing the broadcast radius. We find that WSNs consume more energy in the near-sink area during data collection, while the energy consumption in the far-sink area is low and there is energy remaining. Therefore, the AAS-LBR scheme makes full use of the remaining energy of the far-sink area node to add up to one active slot and increase the broadcast radius. Increasing the broadcast radius can expand the range of one broadcast, so that the number of nodes receiving the code in one broadcast becomes larger, thereby reducing the number of broadcasts needed. On the other hand, it makes the distance of one hop broadcast farther, so that it can spread to the whole network with fewer hops, reducing delays. Adding one active slot can double the time slot of the node receiving code, so that the probability of receiving the code is doubled, which can effectively reduce the number of broadcasts and reduce the delay. We demonstrate that at most only one active slot needs to be added to greatly improve the performance of code dissemination.
(2) An effective code dissemination algorithm based on adding Active Slot joint Larger Broadcast Radius is proposed for duty cycle-based WSNs. The algorithm proposed in this paper significantly improves the performance of code dissemination. Compared with the previous algorithms, the code dissemination algorithm has the following two main improvements: (a) When the broadcast backbone is constructed, the active slot is added to the next hop node with pipeline style in the diffusion path, so that the code dissemination process can be continuously performed without any pause in the proposed scheme. Code can quickly spread to the entire broadcast backbone. (b) For the nodes on the non-broadcast backbone, an active slot is added coincident with its broadcast backbone’ active slot which can fully utilize the broadcast operation of the broadcast backbone to obtain the code, thereby reducing the time required for code dissemination and the number of broadcasts.
(3) Through our extensive theoretical analysis and experiment, we demonstrate that the AAS-LBR scheme proposed in this paper has better performance. Compared to the schemes of previous strategies, our AAS-LBR scheme is superior in all important performance indicators. (a) Compared to previous strategies, the AAS-LBR scheme can reduce the number of broadcasts by 20.0%; (b) the code diffusion delay is reduced by 45.5% compared to previous strategies; (c) the proposed strategy can effectively improve energy utilization by 24.5%. While all major performance factors are improved, its network lifetime is higher than with previous strategies, which was difficult to achieve with the previous strategies.

The rest of the paper is organized as follows: Section 2 reviews related works in comparison with our scheme. Section 3 describes the network model and defines the problem statements of this paper. In Section 4, we give the design of our AAS-LBR scheme for WSNs. The results of the theoretical analysis are given in Section 5. In Section 6, experimental results and comparisons of the AAS-LBR scheme are presented. We conclude this paper in Section 7.

## 2. Related Work

Wireless sensor networks are an emerging technology that has developed rapidly in the past 10 years [49,50,51]. Due to their low price, small size and rich ability to sense data, sensor nodes are deployed in an area of interest to obtain data for various types of applications, thus laying a foundation for the development of the Internet of Things [51,52,53]. Sensor nodes are mainly composed of five main components: processor, battery, memory, communication unit, and sensing unit. Because of the simple hardware, the processing capabilities of the sensor nodes are weak, and the power is limited. These are the main fields of WSNs research, so there are many studies in this area [54,55,56,57]. This section mainly discusses the following main issues related to this paper.

(1) The energy consumption in wireless sensor network data collection has unbalanced characteristics. The most important function of a wireless sensor network is to monitor and perceive certain physical phenomena of interest, or events, so that the data of interest can be obtained in time [44,58]. Since sensor nodes can be deployed on a large scale, continuous and large-volumes of data can be obtained continuously for a long time. With the development of artificial intelligence technology, combined with big data network, Edge network, Cloud computing technology, the basic roles of WSNs have become more prominent [59,60,61]. An important feature of wireless sensor network data collection is the many-to-one data collection feature [48]. In WSNS, *n* sensor nodes and 1 sink are deployed. The sink is the center of the entire network, and all sensor nodes transmit the perceived data to the sink through multi-hop routing [48]. Therefore, in this data collection mode, the data sensed by the nodes in the network must be relayed to the sink through the node within the range of the near 1 hop. Thus, the energy consumption of the nodes in the near-sink hop range is much higher than that in the far-sink area. When the energy consumption of the nodes in the near-sink area is exhausted (called an energy hole), causing the network to die prematurely, no more sensing data cannot be routed to the sink, although there may still be a large number of surviving nodes in the network. According to the work in [14,15,43], when an energy hole occurs, up to 90% of the energy main still remain in the network.

The emergence of energy holes causes the network to die prematurely, which causes serious harm to the network. Therefore, there are many studies on how to overcome the impact of the energy holes to improve the network life. These main lines of research include: (1) Adoption of an optimized broadcast radius r. In general, the larger the broadcasts radius, the greater the number of nodes in a hop range, so that more nodes are responsible for forwarding network data. Obviously, the larger r is, the less data the node bears. The most extreme case is that each node sends directly to the sink, and each node only bears one packet generated by its own node in a data collection period. Although increasing r can reduce the amount of data that the node bears, more r is not always better. Since the energy consumption of transmitting unit bits is proportional to the square of the transmission distance, increasing r will cause the energy consumption of transmitting unit bit data to increase sharply. Therefore, some researchers have given an optimized r to reduce energy consumption in the near-sink area [14,15]. (2) Clustering network. Due to the temporal and spatial correlation between events or physical phenomena perceived by sensor nodes [48], there is some redundancy in the correlation between the perceived data between multiple nodes. Therefore, data aggregation can be performed when multiple data packets meet, thereby reducing the data that needs to be transmitted. A clustered network is a classic data aggregation routing method for data aggregation [26,36,50]. In such a network, the nodes are divided into clusters one by one, and each cluster has a node called cluster head, the other nodes are cluster member. Member node sends its own data to the cluster head, which aggregates the data and routes it to the sink through multi-hop (or single-hop) transmission between cluster heads. The data fusion method can significantly reduce the amount of data that needs to be transmitted in the network, thereby reducing the energy consumption of the nodes. However, the data fusion method only reduces the energy consumption of the nodes, and the energy consumption between the nodes is still unbalanced. The energy consumption of the near sink node is still much higher than that of the far-sink area node. Although the imbalance of energy consumption brings disadvantages to the network, the scheme of this paper can make full use of the imbalance of energy consumption during data collection in the network to improve the performance of code dissemination. In the strategy proposed in this paper, the remaining energy existing in the far-sink area is used as the foundation to improve the performance of code dissemination [6,14].

**Figure 1:**
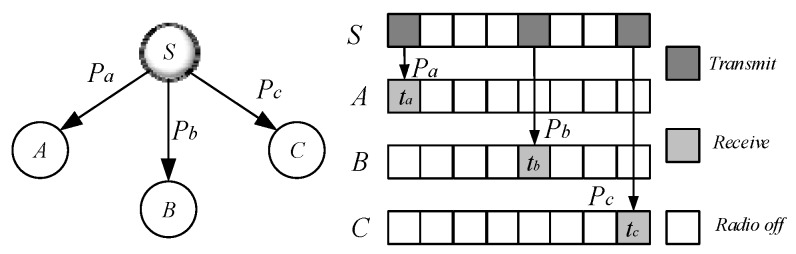
Code dissemination method in loss and low duty cycle-based WSNS.

(2) Code dissemination in non-duty cycle-based WSNs. In non-duty cycle-based WSNs, nodes are always active, although its energy consumption is high, but this is beneficial for code dissemination, because as long as the sender broadcasts, the nodes in its broadcast range can receive the code, so its dissemination speed and complexity are much smaller than in duty cycle-based WSNs. However, at this time, it is necessary to optimize which nodes are selected for broadcasting [62]. If each node that receives the code broadcasts, it will form a broadcast storm, which will consume a lot of energy in the network. Therefore, it is often necessary to construct a Minimum Covering Node Set (MCNS) for the network [59,60]. The nodes in MCNS have the following characteristics: (a) Any two nodes in this set are connected; (b) and all nodes except the nodes in the set can be connected to the nodes in the set by one hop; (c) a minimum number of nodes. Since the MCNS has the above features, it is only necessary to send the code to the node in the MCNS to enable each node in the network to receive the code when the number of broadcasts is small. After the above discussion, the problem of code dissemination has turned into construction of the MCNS. For related research, see the Set-Cover-based Approximation (SCA) scheme in [63].
(3) Code dissemination in duty cycle-based WSNs. Code in duty cycle-based WSNs are very different from that in previous studies. In the duty cycle-based WSNs, the node is active in only one slot among n slots of one cycle, and the other slots are in sleep state. When the sender sends the code in its active slot, not all nodes in its sending range active in this slot, so some nodes may be in sleep state and cannot receive the code. The sender needs to send multiple times in other slots to send the code to all nodes within its radius. As shown in Figure 1, the serial numbers of the active slots selected by node A, B, and C are $t_{a}$, $t_{b}$, $t_{c}$ slot [53], node S wants to pass the code to these three nodes, which need to be sent three times in slots $t_{a}$, $t_{b}$, $t_{c}$ respectively, instead of just broadcasting once like in a non-duty cycle-based WSN. Therefore, the method of code dissemination in such a network is very different from the previous ones. Researchers call such problems the minimum-transmission broadcast (MTB) problem in duty-cycled networks (MTB-DC problem) [53,63]. Obviously, the MCNS method similar to non-duty cycle-based WSNs can also be used to solve such problems. The main steps are as follows: (a) select a part of the node that can cover the entire network, which is called the Dominating Set (DS). (b) Constructing a routing path from the code source node to each node in the Dominating Set, called the broadcast backbone. The broadcast backbone may contain nodes in the non-Dominating Set. After the broadcast backbone is constructed, the code is routed through the nodes on the broadcast backbone. After the nodes on the broadcast backbone receive the codes, the nodes in the Dominating Set broadcast the code to their governed nodes. Since the nodes in the Dominating Set cover all the nodes in the network, this method enables each node in the network to obtain codes. However, the MTB-DC problem is very different from the MTB-non-DC problem. First, in the broadcast backbone route, since the node adopts the duty cycle mode, it must wait for the active slot of its next node to arrive when routing, thus increasing the delay. Secondly, in the non-duty cycle based WSNs, when all the nodes in the broadcast backbone obtain the codes, all the nodes in the network obtain the code. However, in the duty cycle based WSNs, when the nodes in broadcast back-bone obtains the code, some dominated nodes may have not got the code, so the dominator may need to be broadcast several times to complete code dissemination. The above study can be found in [63].

As can be seen from the above research, the main reason for the increase in code dissemination delay and the number of broadcasts is that the node can only perform data operations in one slot among n slots, thus causing the delay of code dissemination to become larger, requiring more broadcast times. Therefore, if the number of active slots of the node increases, the delay of the code dissemination and the number of broadcasts can be effectively reduced. Liu et al. [6] proposed a method to increase the active slot based on the above ideas. However, simply increasing the active slot can reduce the delay and the number of broadcasts, but it will reduce the lifetime. Reference [6] cleverly utilizes the energy consumption imbalance of WSNs in data collection, making full use of the remaining energy of the far-sink area node to increase its active slot. This can effectively reduce the delay and the required number of broadcasts. Although [6] uses the remaining energy to increase the active slot, there are two point that can be further improved as follows: (a) In the method of [6], the activity increase of the nodes is random, and from a statistical point of view, it is inevitable to reduce the delay and broadcast times of code dissemination. However, since the added slots are not randomly allocated and are not effectively allocated, the network performance is not improved very much. For example, when code routing is performed in the broadcast backbone, the active slot of the node is randomly added, which will reduce the delay. Due to the fact the added active slot is not just the next slot of active slot in the upper node, the delay reduction is not large enough, and if the active slot can be added just to the next slot of the active slot of the sender node, then, after the sender receives the code, the code can be forwarded to the receiver in the next slot, which can greatly reduce the delay. (b) In fact, there is no need to increase multi-active slots as in [6]. In this paper, we have confirmed that only one active slot for nodes can achieve high performance.

Yu [17] also proposed another method called the ABRCD scheme to improve the code dissemination performance. The ABRCD scheme makes full use of the remaining energy of the far-sink area nodes to increase the broadcast radius of the nodes from another view. The advantage of increasing the broadcast radius is very obvious: because the broadcast radius is increased, the range in which the node broadcasts is increased. Due to the fact the broadcast range is increased, the number of nodes receiving the code in the broadcast range increases, and the number of nodes that receive the code once increases, so that the number of broadcasts can be reduced. On the other hand, as the broadcast radius increases, the number of nodes required to construct the MCNS decreases, so the number of nodes required to construct the broadcast backbone is reduced, thereby reducing the delay required for the broadcast backbone route. Therefore, the ABRCD scheme can effectively reduce the delay and number of broadcasts of code dissemination. However, there are some room for improvement in their methods. In their method, no attention is paid to the adjustment of the slot.

(4) Code spread in loss and low duty cycle based WSNs. The previous discussion is based on the assumption that wireless communication is reliable. In other words, the sender only needs to send once, then the receiver can successfully receive the code. However, in actual WSNs, due to the attenuation of the wireless channel and the complexity of the transmission environment, the receiver receives the code successfully with a certain probability such as p. Chen et al. [53] proposed a strategy for code dissemination in unreliable WSNs. As shown in Figure 1, node S is ready to spread code to node A, B, C and its transmission success rates are $P_{a}$, $P_{b}$, $P_{c},$ respectively. Therefore, node S send code in $t_{a}$, $t_{b}$, $t_{c}$ slot, and node A, B, C respectively receive data in these three active slots [53].

Due to the unreliability of wireless communication, node S may not be able to send successfully once. Since the probability of successful transmission is p, the expected transmissions number is 1/p for receiver get code successful. Therefore, if the transmission fail, node S will send again in the active slot of in the next cycle. The above process continue until node S send successfully [53].

**Figure 2:**
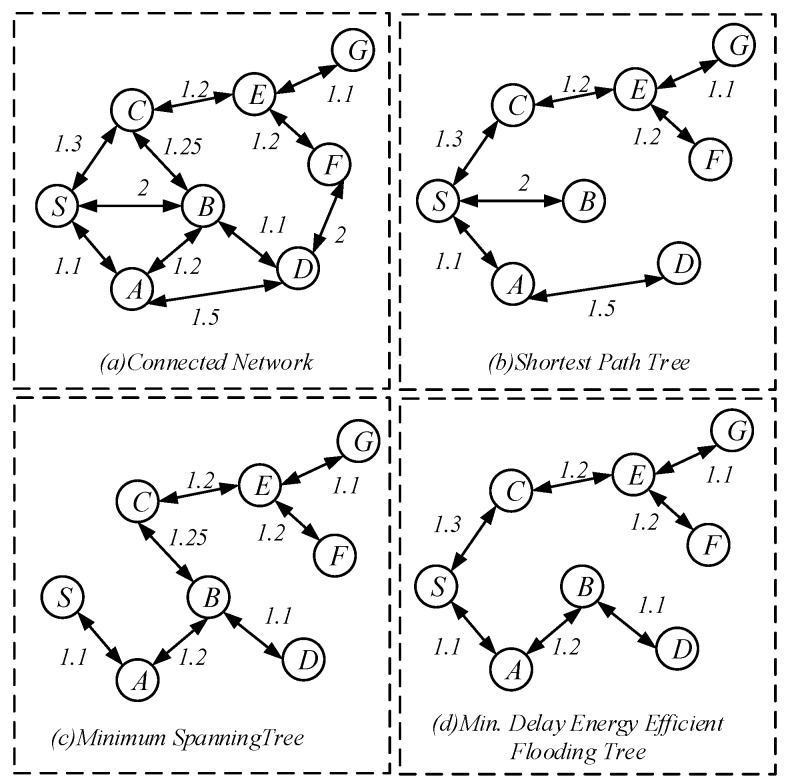
3 different code dissemination strategy diagrams.

For the spread of code throughout the network, [53] compared several classical dissemination strategies. Figure 2a gives the topology of the network. The edges in the figure indicate that there is a communication link between the nodes, and the weight on the side indicates the expected number of transmissions required to successfully send the code, which is the 1/p. At this time, to make the code spread to the minimum delay of each node in the network, the shortest path algorithm can be adopted as shown in Figure 2b, and to minimize the number of transmission broadcast, the routing path formed by the minimum spanning tree shown in Figure 2c can be used. Chen et al. [53] proposed a delay energy efficient code dissemination algorithm to obtain tradeoff between delay and energy efficiency [64,65].

## 3. System Model

### 3.1. System Network Model

In this paper, the same network as in [6,14,17] is used, and G=(V,E) is used to represent the wireless sensor network, where V represents the set of sensor nodes in the network, and E is the edge set. If the Euclidean distance between nodes u and v is within its communication range, then the edge (u, v) belongs to E. $v_{i}$ represents the i-th node in the network, i∈{1, 2, 3 …N}, and the total number of nodes is N. The nodes in the network are given a unique ID. The radius of the network in G is R, and the radius of the broadcast of each sensor node is r. The sensor nodes are powered by batteries with limited energy, and the energy of the sink is infinite.

**Figure 3:**
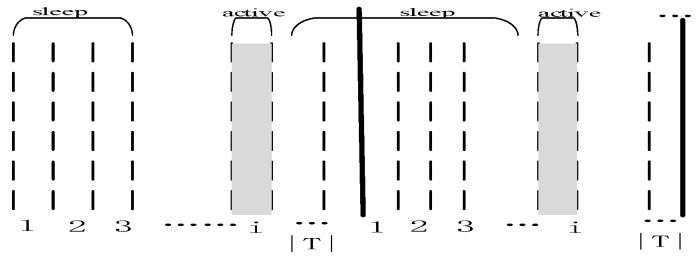
Example of periodical wake up of node *v*.

The network is a duty cycle-based WSN. The node works in periodic sleep/awake rotation mode. The length of each cycle is T, consisting of n slots, the number of the slot is {0, 1, 2, 3, …, n−1} (see Figure 3). The period length is represented by |T|, which is the number of slots in the period, n=|T|. Thus, the series of slots can also be expressed as {0, 1, 2, 3, …, |T|−1} slots. The node selects one or more slots from the n slots as the active slot, and the other slots sleep. The network adopts the asynchronous working mode, and each node in the network independently selects its own sleep slot and active slot. A slot is long enough to accept information from other nodes and send information to other nodes and the node can only receive data in the active state, but the node can send data in any slot when it needs to send code by turning itself into an awake state. 

### 3.2. The Energy Consumption Model

The energy consumption of the paper is the same as in [49]. The energy consumption of a node includes the following three aspects: (1) energy consumed by transmitting data; (2) energy consumed by receiving data; and (3) low-power listening (LPL), which refers to the energy consumption when the node does not send data or receive data in an awake status. Obviously, the energy consumption of the node in the LPL state is low. The energy consumption E of the node can be expressed by Equation (1). In Equation (1), $E_{t},E_{r},E_{LPL}$ and $E_{s}$ represent the energy consumption of the node transmitting data, receiving data, LPL status, and node sleep, respectively:

(1) $$E=E_{t}+E_{r}+E_{LPL}+E_{s}$$

The energy consumption of a node can be expressed by the following formula:

(2) $$E=\omega_{r}Q_{r}+\omega_{t}Q_{t}+E_{LPL}+E_{s}$$

where $\omega_{r}$, $\omega_{t}$ represent the average energy consumption of a packet received by the node, and the average energy consumption of sending a packet; $Q_{t}$ and $Q_{r}$ respectively represent the data sent by the node and the received data. The energy consumption parameters of this paper are listed in Table 1 which can found in [28].

However, in this paper, the broadcast radius of the node is variable, so the transmission power corresponding to the node is variable. Obviously, if the transmission power of the node is large, the broadcast radius of the node is large. Fortunately, many current wireless sensor nodes have different power levels that can be adjusted. For MICA2, there are 26 transmission power levels of −20 dBm to 5 dBm, as shown in Table 2. Thus, when a node uses a different broadcast radius, its transmission power can be obtained from Table 2.

### 3.3. Problem Statement

The purpose of this paper is to reduce the number of broadcasts, delays, and improve network life and energy utilization. The specific discussion is as follows:

**Definition** **1.** *Code dissemination delay. Code dissemination delay refers to the difference between the time when the code starts to spread the code and the time when the last node in the network receives the code.*

(1) Minimize code dissemination delay. Obviously the first goal of this paper: Minimize code dissemination delay can be expressed by the following Equation (3):
  (3) $$Min\left(D\right)=min\left(max\left(T_{i}\right)-T_{s}\right)$$
  where $T_{i}$ indicates the time at which node i obtains code, so max$\left(T_{i}\right)$ indicates the time at which the code node was last obtained. $T_{s}$ indicates the time at which the sink initiates code dissemination, and D indicates the maximum delay of code dissemination. Therefore, the goal of optimization is to minimize the delay of the last code node in the network.
(2) Minimum−transmission broadcast (MTB). The second optimization target of the strategy design is MTB, which makes the minimum number of broadcasts required from code sink to transmission to all nodes in the network. $B_{i}$ is the set of transmission slots of node $v_{i}$, and $\left|B_{i}\right|$ represents the number of broadcasts of node $v_{i}$. Then the minimum-transmission broadcast is as shown in Equation (4) below:
  (4) $$min\left(B\right)=min\sum_{i\in \left\{1,2,3\ldots N\right\}}\;\left|B_{i}\right|$$
(3) Maximize network life. The lifetime of the entire network can be defined as the time until the first node dies. Since the death of the first node may affect the connectivity of the network, the function of the entire network is impaired and the requirements for normal operations cannot be reached. Therefore, the network life cycle in this paper is the time when the first node dies. $E_{0}$ represents the initial energy of the node, and $e_{i}$ represents the average energy consumption of the node $v_{i}$ in a period T. The maximum lifetime of the network L is to maximize the lifetime of the node with the smallest lifetime in the network:
  (5) $$max\left(L\right)=max\left(min\left(E_{0}/e_{i}\right)\right)$$
(4) Maximize energy utilization. U indicates the ratio of the energy consumed by the entire network to the initial energy of the entire network. $E_{e,i}$ indicates the energy consumption of node $v_{i}$ when the network dies. Then the energy utilization can be maximized as shown in Equation (6):
  (6) $$max\left(U\right)=max\left(\frac{\sum_{i=1}^{N}E_{e,i}}{NE_{0}}\right)$$

Obviously, this can achieve the optimization goal of this paper as shown in Equation (7):

(7) $$\{\begin{array}{l} Min\left(D\right)=min\left(max\left(T_{i}\right)-T_{s}\right)\\ \;min\left(B\right)=min\sum_{i\in \left\{1,\;2,\;3\;\ldots N\right\}}\;\left|B_{i}\right|\\ \;max\left(L\right)=max\left(min\left(E_{0}/e_{i}\right)\right)\\ \;max\left(U\right)=max\left(\frac{\sum_{i=1}^{N}E_{e,i}}{NE_{0}}\right) \end{array}$$

## 4. The Design of AAS-LBR Scheme

### 4.1. The Research Motivation

The research motivation mainly comes from our observation of the following laws of sensor network information dissemination, and make full use of these rules to reduce the number of broadcasts and delay of code dissemination. 

(1) Increasing the node’s active slot can effectively reduce the number of broadcasts of the codes dissemination and delay.

**Figure 4:**
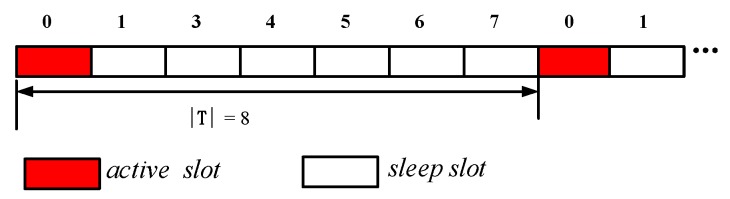
The duty cycle of node.

**Figure 5:**
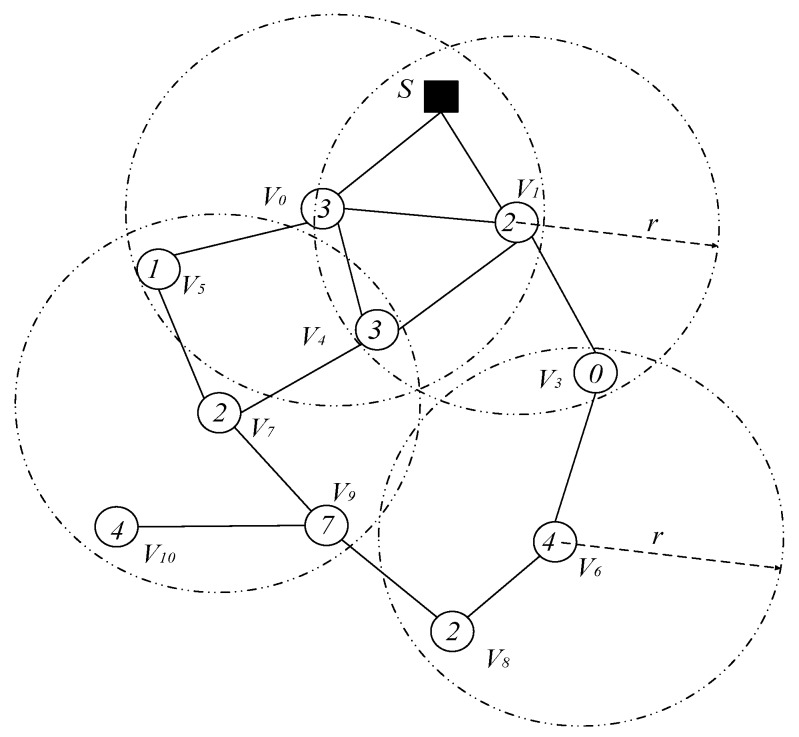
The codes dissemination in duty cycle based wireless sensor network.

The node’s duty cycle in wireless sensor networks can be illustrated as in Figure 4. The working period of the node is T, and one working cycle is composed of n slots. The number of slots in a duty cycle is represented by |T|, i.e., |T| = n. Slots are numbered starting from 0 to |T|−1, i.e., {0, 1, 2, …, |T|−1}. The sensor node selects one of the time slots as an active slot. In this slot, the node is in the awake state and can perform data receiving and sending operations. When the node is in other slots except the active slot, the node is in a sleep state, and data is not received and sent at this time. As shown in Figure 4, the number of slots in a duty cycle of a node |T| = 8, the node selects slot 0 as the active slot (see the node $v_{3}$ in Figure 5), where the slot can receive and send data. In other slot sleeps, data cannot be received and sent.

Due to the above-mentioned duty cycle working mode, the number of broadcasts of the codes dissemination and the delay are relatively large. As shown in Figure 5, the sink S has codes, and needs to propagate the codes to each node in the network. The number in the node in the figure indicates the serial number of the active slot selected by the node. For the nodes $v_{3}$ and $v_{4}$ in Figure 5, the active slots selected are 0, 3, respectively. The traditional codes dissemination is performed for such a network as follows: since the active slots selected by $v_{3}$ and $v_{4}$ are 0, 3, respectively, if the node $v_{1}$ is responsible for the code broadcast of node $v_{3}$, $v_{4}$, then node $v_{1}$ must broadcast each of the two nodes in slot 0, 3 to make these two nodes receive the codes. This leads to more broadcasts.

**Figure 6:**
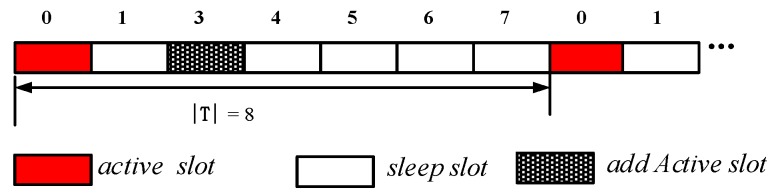
The time slot with an added active time slot.

However, if node $v_{3}$ adds an active slot, the performance of its code dissemination will be greatly improved. Figure 6 shows the addition of an active slot to node $v_{3}$, where slot 3 is also an active slot. 

After adding an active slot, node $v_{1}$ only needs to broadcast once in slot 3, and nodes $v_{3}$ and $v_{4}$ can receive the code, which can reduce one broadcast operation. If most nodes in the network can add one active slot, the number of broadcasts required for the entire network code dissemination can be greatly reduced. Therefore, we can conclude that increasing the active slots of the node can effectively reduce the number of broadcasts required.

On the other hand, adding an active slot to a node can also significantly reduce the delay of the code dissemination. In the above example, since the active slot of node $v_{1}$ is 2. Thus, node $v_{1}$ will get the code from sink S or node $v_{0}$ in slot 2. Node $v_{1}$ can only propagate code forward after getting code. If node $v_{3}$ does not add an active slot, node $v_{1}$ can broadcast to node $v_{4}$ when it gets the next slot of the code, slot 3, at this time, the delay that node $v_{1}$ propagates to $v_{4}$ in one slot. However, the active slot of node $v_{3}$ is 0, so node $v_{1}$ can only propagate code to node $v_{3}$ in slot 0 of the next cycle (see Figure 5). Therefore, the delay for node $v_{1}$ to propagate data to $v_{3}$ is six slots, and if node $v_{3}$ adds an active slot in slot 3, then node $v_{1}$ broadcasts once to make node $v_{3}$, $v_{4}$ get code. At this time, the delay that node $v_{1}$ propagates to these two nodes is one slot, which can reduce the delay by five slots. 

(2) Increasing the broadcast radius of the node can reduce the number of broadcasts and delay.

**Figure 7:**
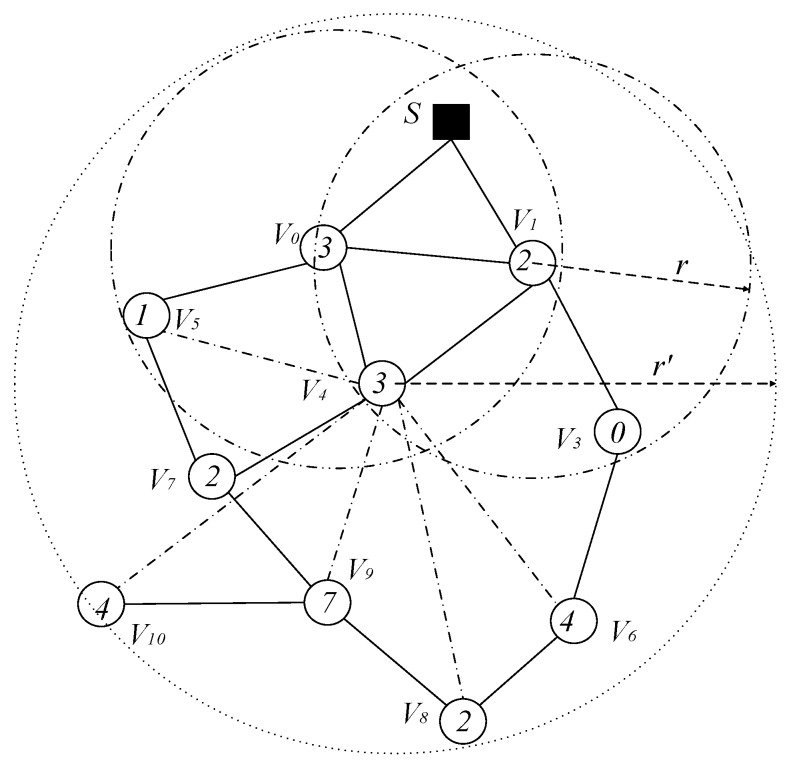
The network topology after increasing the radius.

When the broadcast radius of node is r, the network topology is shown in Figure 5. If the broadcast radius of nodes $v_{3},\;v_{4},\;v_{5},\;v_{6},\;v_{7}\;\mathrm{and}\;v_{8}$ can be increased from r to $r^{\prime }$, then the network topology is as shown in Figure 7. 

The propagation process and performance analysis of the codes dissemination under these two different broadcast radius are as follows: we use (v,$\left\{S_{v}\right\}$) to indicate that node v performs a broadcast operation in time slot $S_{v}$. When the broadcast radius is not increased, the series of broadcast operations required for code to propagate from sink S to each node of the network is: {(*S*, 2), ($v_{1}$, {0, 3}), ($v_{0}$, 1), ($v_{3}$, 4), ($v_{7}$, 7), ($v_{9}$, {2, 4})}. The number of broadcasts required is eight, and the delay is 21 slots.

After the radius increase, the series of broadcast operations required for code to propagate from sink S to each node of the network are: {(s, 2), ($v_{1}$, {0, 3}), ($v_{0}$, 1), ($v_{4}$, {2, 4, 7})}. In the case the broadcast radius is expanded, the number of broadcasts required is six and the delay is 11 slots. Compared with the strategy of not expanding the broadcast radius, the number of broadcasts was reduced by two times, and the delay was reduced by 10 slots, which reduces the number of broadcasts by 25.0% and the delay by 47.6%.

(3) Simultaneously increasing the active slot of the node and increasing the broadcast radius of the node can further reduce the number of broadcasts and the delay.

**Figure 8:**
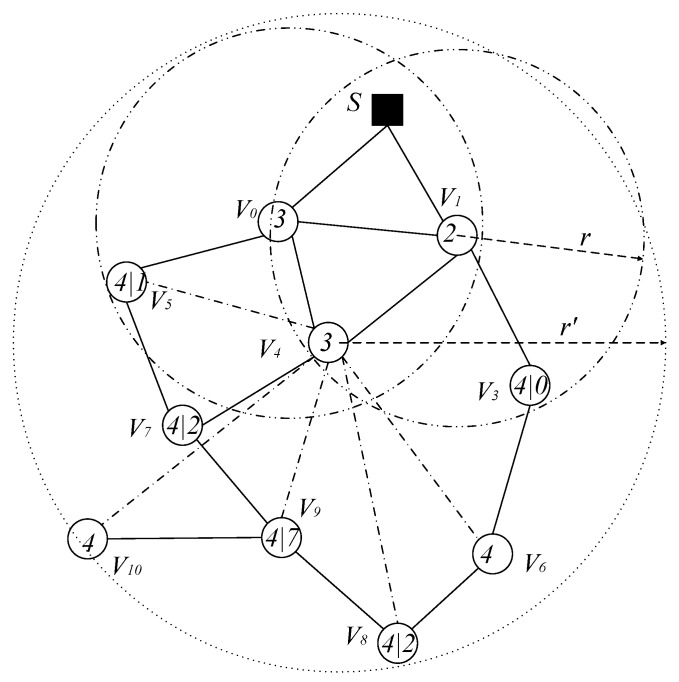
The network topology and slot status after increasing active and increasing radius.

Obviously, from the above analysis, it can be seen that increasing the node’s active slot and simultaneously expanding the node’s broadcast radius can further reduce the number of broadcasts and delay. We illustrate this by still using the above example. As shown in Figure 8, if node $v_{3},\;v_{5},\;v_{7},\;v_{8}\;\mathrm{and}\;v_{9}$ add an active slot in slot 4 and we increase the broadcast radius of node $v_{3},\;v_{4},\;v_{5},\;v_{6},\;v_{7}\;\mathrm{and}\;v_{8}$ from r to r′, at this time, the codes dissemination operation is as shown below: Sink S passes the code to $v_{1}$ in slot 2, $v_{1}$ broadcasts to $v_{0}$ and $v_{4}$ in slot 3, and $v_{4}$ broadcasts to all nodes that have not received code in the next slot 4. Thus, starting from the 2nd slot and ending at the 4th slot, only two delays of the slot are needed, and the number of broadcasts including Sink S only needs three broadcasts. Compared with no optimization measures, the optimized broadcast frequency and delay are reduced by 62.5% and 90.5%, respectively.

(4) There is a large amount of remaining energy in the wireless sensor network to increase the active slot and expand the broadcast radius without affecting the network lifetime.

From the previous discussion, it can be seen that increasing the active slot of the node and increasing the broadcast radius of the node can effectively reduce the number of broadcasts and the delay, and the improved network performance with the combination of the two is very high. However, a key problem here is that increasing the active slot and increasing the broadcast radius all need to consume the energy of the node, and the energy is very limited in the wireless sensor network. Increasing the active slot and broadcast radius require energy, which may affect the network lifetime, so in the previous strategy, this method of increasing the active slot and increasing the broadcast radius is not used. 

However, after careful analysis, we found that increasing the active slot and increasing the broadcast radius in the wireless sensor network will not affect the network lifetime. Wireless sensor networks collect data most of the time when sensor nodes sense events in the surrounding environment, or other data of interest, and then send them to the sink via multi-hop routing. Therefore, the sink is the center of the entire network data collection, and the amount of data moved within the near-sink area is far higher than other areas which is called hotspots. The sensor network dies early due to the high energy consumption of the hotspots nodes. After the nodes in the hotspots area die, a so-called “energy hole” is formed. After the “energy hole” is formed, the data of other nodes in the network cannot be routed to the sink, thus causing the entire network to die. According to research, when a network dies early due to the “energy hole”, the energy still remaining can be as high as 90% [63]. Therefore, this article is inspired by the above phenomenon. If the active slot and the broadcast radius are simultaneously increased in the area where the energy is surplus in the far-sink area, this will reduce the number of broadcasts and delay without affecting life. It can be illustrated through a concrete example that the wireless sensor network has a large amount of remaining energy that can be used to increase the active slot and increase the broadcast radius without reducing the network lifetime.

**Figure 9:**
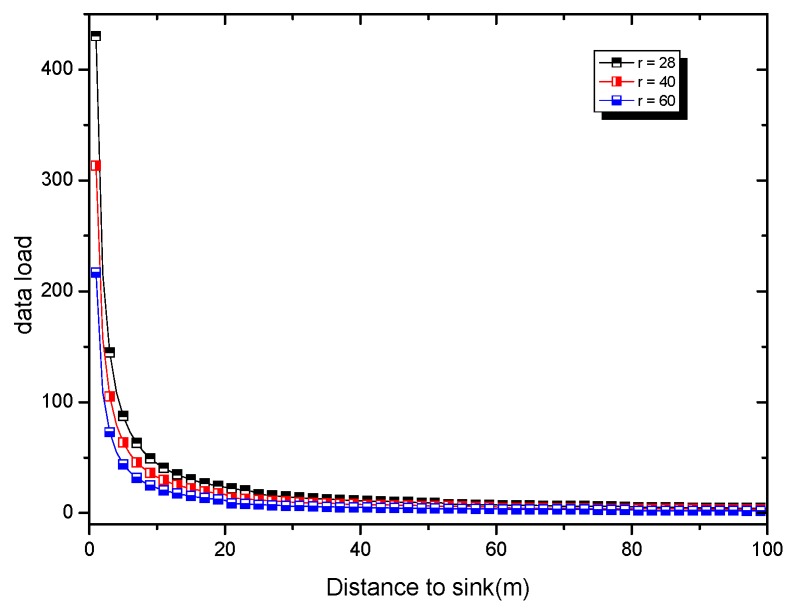
The amount of data that the node assumes decreases as the distance from the sink node decreases.

**Figure 10:**
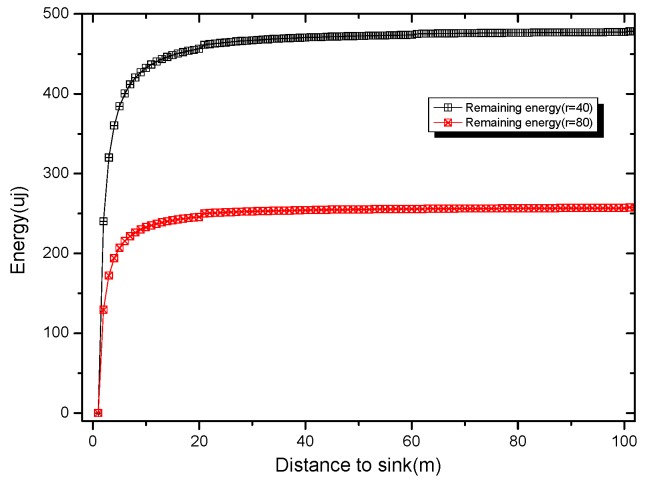
The remaining energy of the node can support increasing the active slot and increasing the radius.

In Figure 9, the data volume of the near-sink area and the far-sink area is shown. It can be seen that the near-sink area bears much more data than the far-sink area. Figure 10 gives the remaining energy in different areas of the network. As can be seen from Figure 10, there is a lot of energy left in the far-sink area. We can use this part of the remaining energy to increase the active slot and increase the broadcast radius, thus effectively improving the code dissemination performance.

Based on the above analysis, we propose the Adding Active Slot joint Larger Broadcast Radius (AAS-LBR) scheme. The core of the AAS-LBR scheme is how to make full use of the remaining energy in the far-sink area to increase the active slot of the node, expand the broadcast radius of the node, and rationally select the active slot to construct an optimized code dissemination strategy, thus optimizing the performance of code dissemination.

### 4.2. The AAS-LBR Scheme

The main points of the design of Adding Active Slot joint Larger Broadcast Radius (AAS-LBR) scheme are as follows:

(1) Calculate the nodes that can increase the active slot according to the energy consumption of the nodes, then these nodes can add one active slot (although some nodes do not necessarily need to increase the active slot). If there is still remaining energy, then calculate the remaining energy that can increase the broadcast radius of the node.
(2) Select a Minimum Covering Node set (MCNS) based on the network topology with the added broadcast radius but without increasing the active slots. MCNS can cover every node in the network, so it only needs to spread the code to each node of the MCNS, and then the node on the MCNS is responsible for code dissemination of its governed node, so that the code can be efficiently spread to the entire network. After selecting MCNS, it is necessary to route the code from the sink to each node in the MCNS, which is to construct the broadcast backbone. However, in the AAS-LBR scheme, constructing the broadcast backbone has greatly improved the previous strategy. In the AAS-LBR scheme, the node in the far-sink area can be incremented by one active, and the active slot can be added to any slot in the node. Therefore, when constructing the broadcast backbone, it is possible to construct a broadcast backbone with the smallest delay. That is to say, when constructing the broadcast backbone, each node can increase the added active slot to the next slot of the current node’s active slot when selecting the next hop node. In this way, after the current node receives the code, it can propagate forward in the next consecutive active slot, thus reducing the delay of constructing the broadcast backbone to a minimum.
(3) Code dissemination stage. Since MCNS has covered the entire network, therefore, after the nodes on the broadcast backbone get the codes, the nodes in the MCNS only need to broadcast the code to the nodes covered by it. In the AAS -LBR scheme, different from the previous strategy, since the active of the node can be added, we will select the active slot of the node to be the same as one of the broadcast slots of the node in the MCNS. In this way, in MCNS, when the broadcast backbone is completed, the node that can increase the active slot has already obtained the code. Therefore, it is not necessary to perform code dissemination for the dominated node as in the previous strategy. This reduces the number of broadcasts and greatly reduces the delay.

#### 4.2.1. The Add Active Slot and Increased Broadcast Radius Calculation

This section mainly calculates the energy consumption of the network to determine whether the nodes at different distances from the sink can increase the active energy by one active slot (the AAS-LBR scheme only requires the node to add up to one active slot), and then calculate whether there *i* remaining energy to expand the broadcast radius of the node.

According to Ref. [6], in a network with a network radius R, each node generates a data packet in a round of data collection and transmits it to the sink using the shortest route algorithm. The amount of data carried by the node far from sink xm is as follows:

(8) $$D_{x}=\left(z+1\right)+\frac{z\left(z+1\right)r}{2x}$$

According to the above conclusion, the probability that the nodes in the network generate data is γ. Therefore, the amount of data carried by the node far from sink xm is as follows:

(9) $$Q_{r}^{x}=\left(\left(z+1\right)+\frac{z\left(z+1\right)r}{2x}\right)\gamma$$

The amount of data sent is the amount of data received plus the amount of data generated by itself:

$$Q_{t}^{x}=Q_{r}^{x}+\gamma$$

The energy consumption $\omega_{t}^{x}$ of sending a packet is:

(10) $$\omega_{t}^{x}=\varepsilon_{t}S_{d}+\frac{\left(1-\varrho \right)T}{2\left(S_{p}+S_{al}\right)}\left(\varepsilon_{t}S_{p}+\varepsilon_{r}S_{al}\right)$$

The energy consumption for receiving a packet is:

(11) $$\omega_{r}^{x}=\varepsilon_{r}S_{d}+\left(\varepsilon_{r}S_{p}+\varepsilon_{t}S_{al}\right)$$

The energy consumption of the corresponding LPL operation can be expressed as:

(12) $$E_{LPL}^{x}=\varepsilon_{r}\varrho +\varepsilon_{s}\left(1-\varrho \right)-\tau_{t}^{x}-\tau_{r}^{x}$$

where $\tau_{t}^{x}$ can be expressed as:

(13) $$\tau_{t}^{x}=\left\{\varepsilon_{s}\left[\frac{\left(1-\varrho \right)T}{2}+S_{p}+S_{al}\right]+\varepsilon_{r}S_{p}\right\}\frac{Q_{t}^{x}}{T}$$

$\tau_{r}^{x}$ is represented by the following formula:

(14) $$\tau_{r}^{x}=\left[\left(S_{al}+S_{d}\right)\varepsilon_{s}+\varepsilon_{r}S_{p}\right]\frac{Q_{r}^{x}}{T}$$

The parameters in Equations (9)–(14), $\varepsilon_{t}$, $\varepsilon_{r}$ and $\varepsilon_{s}$ represent transmission power, received power and sleep power, respectively; ϱ represents the duty cycle of the node; $S_{d}$, $S_{al}$ and $S_{p}$ represent Packet duration, Ack window duration and Preamble duration, respectively. The corresponding values of the parameters are from Table 1.

**Theorem** **1.** *In a sensor network with radius*R*, if the node uses a broadcast radius of*r*, the energy consumption of the near-Sink area is*$e_{max}$*, and the energy consumption of the area node with a distance sink of*x*is*$e_{x}$*. After adding an active slot to the remaining energy of the node, the duty cycle of the node is*$\varrho^{\prime }$*, and its energy consumption is*$e_{x}^{\prime }$*, then*x*satisfies the following conditions:*

(15) $$x\geq \frac{r\gamma Z\left(Z+1\right)\left[\left(C+D\right)-\frac{A+B}{T}\right]}{2\left(e_{max}-E_{s}-\varepsilon_{r}\varrho^{\prime }-\varepsilon_{s}\left(1-\varrho^{\prime }\right)+\frac{A\gamma }{T}-D\gamma \right)-\gamma \left(Z+1\right)\left[\left(C+D\right)-\frac{A+B}{T}\right]}$$

*where A =*$\varepsilon_{s}\left[\frac{\left(1-\varrho^{\prime }\right)T}{2}+S_{p}+S_{al}\right]+\varepsilon_{r}S_{p}$, *B =*$\left(S_{al}+S_{d}\right)\varepsilon_{s}+\varepsilon_{r}S_{p}$, *C =*$\varepsilon_{r}S_{d}+\left(\varepsilon_{r}S_{p}+\varepsilon_{t}S_{al}\right)$, *D =*$\varepsilon_{t}S_{d}+\frac{\left(1-\varrho^{\prime }\right)T}{2\left(S_{p}+S_{al}\right)}\left(\varepsilon_{t}S_{p}+\varepsilon_{r}S_{al}\right),\;E_{s}$*is the energy consumption of the node in sleep state.*

**Proof.**  In a sensor network with a radius R, the energy consumption of the near-sink area is $e_{max}$, and the energy consumption of the area node with a distance sink of x is $e_{x}$, then the energy surplus of the node is $e_{max}-e_{x}$. If the energy remaining of the node can be increased by one active slot, and the increased energy consumption is $e_{x}^{\prime }$, $e_{x}^{\prime }-e_{x}$ is consumed more than no time slot is added, and this part of the energy comes from $e_{max}-e_{x}$. So the condition for adding an active slot is:

$$\begin{array}{l} e_{max}-e_{x}\geq e_{x}^{\prime }-e_{x}\\ \Rightarrow e_{max}\geq \omega_{r}^{x}Q_{r}^{x}+\omega_{t}^{x}Q_{t}^{x}+E_{LPL}^{x}+E_{s}\\ \Rightarrow e_{max}\geq [\varepsilon_{r}S_{d}+(\varepsilon_{r}S_{p}+\varepsilon_{r}S_{al})]Q_{r}^{x}+[\varepsilon_{t}S_{d}+\frac{\left(1-\varrho^{\prime }\right)T}{2\left(S_{p}+S_{al}\right)}(\varepsilon_{t}S_{p}+\varepsilon_{r}S_{al})](Q_{r}^{x}+\gamma )+\varepsilon_{r}\varrho^{\prime }+\\ \;\varepsilon_{s}(1-\varrho^{\prime })-\{\varepsilon_{s}[\frac{\left(1-\varrho^{\prime }\right)T}{2}+S_{p}+S_{al}]+\varepsilon_{r}S_{p}\}\frac{\left(Q_{r}^{x}+\gamma \right)}{T}-[(S_{al}+S_{d})\varepsilon_{s}+\varepsilon_{r}S_{p}]\frac{Q_{r}^{x}}{T}+E_{s}\\ \Rightarrow e_{max}-E_{s}-\varepsilon_{r}\varrho^{\prime }-\varepsilon_{s}\left(1-\varrho^{\prime }\right)-D\gamma +\frac{A\gamma }{T}\geq \left(C+D-\frac{A+B}{T}\right)Q_{r}^{x}\\ \;\Rightarrow \frac{e_{max}-E_{s}-\varepsilon_{r}\varrho^{\prime }-\varepsilon_{s}\left(1-\varrho^{\prime }\right)-D\gamma +\frac{A\gamma }{T}}{C+D-\frac{A+B}{T}}\geq \left(\left(z+1\right)+\frac{z\left(z+1\right)r}{2x}\right)\gamma \\ \;\Rightarrow \frac{2\left(e_{max}-E_{s}-\varepsilon_{r}\varrho^{\prime }-\varepsilon_{s}\left(1-\varrho^{\prime }\right)-D\gamma +\frac{A\gamma }{T}\right)}{r\gamma z\left(z+1\right)\left(C+D-\frac{A+B}{T}\right)}-\frac{z+1}{z\left(z+1\right)r}\geq \frac{1}{x}\\ \;\Rightarrow x\geq \frac{r\gamma Z\left(Z+1\right)\left[\left(C+D\right)-\frac{A+B}{T}\right]}{2\left(e_{max}-E_{s}-\varepsilon_{r}\varrho^{\prime }-\varepsilon_{s}\left(1-\varrho^{\prime }\right)+\frac{A\gamma }{T}-D\gamma \right)-\gamma \left(Z+1\right)\left[\left(C+D\right)-\frac{A+B}{T}\right]} \end{array}$$

□

**Figure 11:**
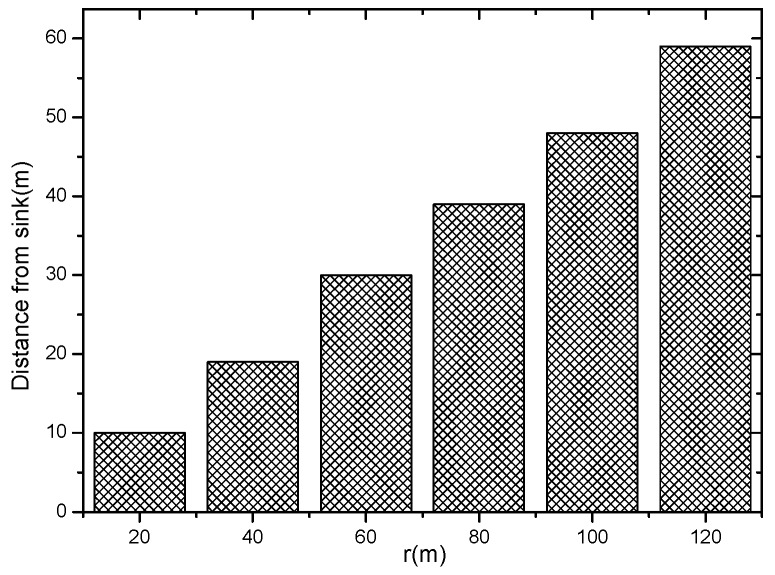
Node that can add a time slot and is closest to the sink.

Figure 11 shows that when the initial broadcast radius takes different values, the distance sink obtained is closest and can increase the distance of an active slot, according to Theorem 1. As can be seen from Figure 11, if the broadcast radius of the network is r, the nodes outside the sink r/2 can be added with one active slot. If the network radius R=κr, the nodes in the area of the network $\left(\kappa^{2}-1/4\right)/\kappa^{2}$ can add 1 active slot without affecting the network lifetime. If κ = 8, more than 99.6% of the nodes in the network can add one active slot and if κ = 10, then up to 99.75% of the nodes can increase the active slot. It can be seen that the remaining energy of most nodes of the sensor network can satisfy the condition of adding an active slot.

According to Theorem 1, the energy consumption $e_{slot}$ of adding an active slot for the remaining nodes of energy is expressed as:

(16) $$\begin{array}{c} e_{slot}=\frac{\left(\varrho -\varrho^{\prime }\right)T}{2\left(S_{p}+S_{al}\right)}\left(\varepsilon_{t}S_{p}+\varepsilon_{r}S_{al}\right)Q_{t}^{x}+\omega_{r}^{x}\;Q_{r}^{x}+\varepsilon_{r}\left(\varrho^{\prime }-\varrho \right)+\varepsilon_{s}\left(\varrho -\varrho^{\prime }\right)-\frac{\left(\varrho -\varrho^{\prime }\right)\varepsilon_{s}TQ_{t}^{x}}{2T}-\\ \tau_{r}^{x} \end{array}$$

In Theorem 1, a node that satisfies adding an active slot may still have remaining energy after adding an active slot. In the AAS-LBR scheme, this part of the energy is used to adjust the broadcast radius of the node. The way to adjust the broadcast radius is as follows: 

The symbols used in the algorithm mean the following: Considering the sensor network with radius R, $k_{0}$ is the initial energy transmission power level used by the network. The transmission power level of the node is shown in Table 2. $x_{0}$ indicates the nearest node distance from the sink. $e_{x}\left(k_{0}\right)$ indicates the energy consumption of the node using the $k_{0}$ power level at a distance *x* from the sink. $q_{x}\left[k\right]$ indicates the transmission power of the k power level at a distance *x* from the sink. $r_{x}\left[k\right]$ represents the transmission radius of the *k*th power level at a distance *x* from the sink. Since the $q_{x}\left[k\right]$ variable has a correspondence with the $r_{x}\left[k\right]$, knowing any one variable can obtain another variable by querying Table 2.

**Figure 12:**
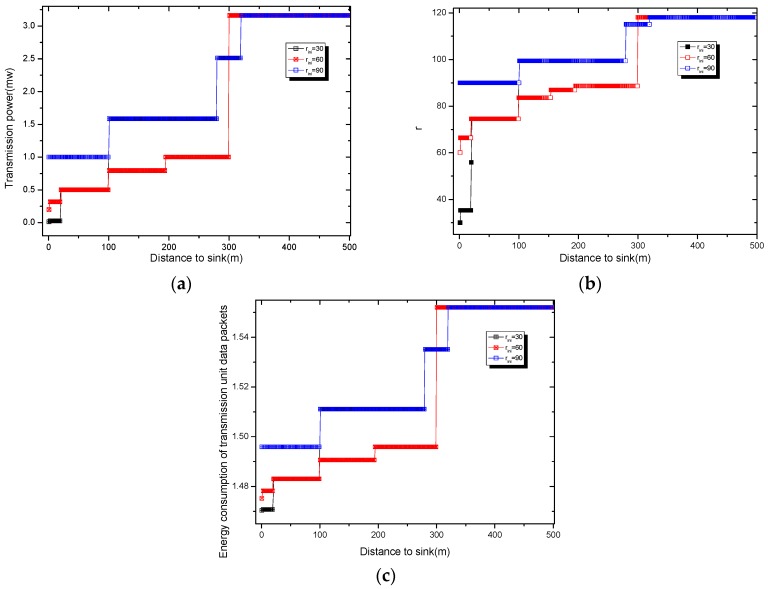
(**a**) Transmission power at different distances from sink; (**b**) broadcast radius at different distances from sink; (**c**) energy consumption of transmission unit data packets.

The idea of the algorithm is as follows: The energy transmission power used by all nodes in the network before the start of the algorithm is $k_{0}$. The energy consumption of the nearest node is the maximum $e_{max}=e_{x_{0}}\left(k_{0}\right)$. According to Theorem 1, the energy consumption of adding an active slot to the node with energy remaining is $e_{slot}$, and then the energy consumption of the node is $e_{x}^{\prime }=e_{x}\left(k_{0}\right)+e_{slot}$. If the residual energy $e_{max}-e_{x}^{\prime }>0$, it means that this part of the energy can be used to expand the broadcast radius. At this time, we increase the transmission power by one level $q_{x}\left[k_{cur}+1\right]$ according to Table 2, and $k_{cur}$ indicates the transmission power level currently used by the node. The corresponding broadcast radius is expanded to $r_{x}\left[k_{cur}+1\right]$, and the energy consumption after the change of the broadcast radius is $\;e_{x}^{\prime \prime }$. If $e_{x}^{\prime \prime }$ > $e_{max}$, it means that the energy after expanding the radius exceeds the energy consumption of the node with the largest energy consumption. We need to adjust the broadcast radius of the node without affecting the network life. At this time, the transmit power of the node is still the transmit power whose last energy consumption does not exceed $e_{max}$, and the broadcast radius remains the same as $r_{x}\left[k_{cur}\right]$. If $e_{x}^{\prime \prime }$ = $e_{max}$, the broadcast radius under this transmit power is exactly as expected, and the broadcast radius of the node is $r_{x}\left[k_{cur}+1\right]$; If $e_{x}^{\prime \prime }$ < $e_{max}$, it indicates that there is still energy remaining after expanding the broadcast radius of the node. This part of energy can still be used to expand the broadcast radius of the node. Repeat the above procedure until the node can find the corresponding maximum broadcast radius whose energy consumption does not exceed $e_{max}$. Algorithm 1 describes the method of calculating the broadcast radius of nodes at different distances from sink. Figure 12a shows the transmission power at different distances from the sink obtained according to Algorithm 1 in the AAS-LBR scheme. It can be seen from Figure 12a that when the initial radius $r_{ini}$ = 90, the transmission power of the far-sink node can be increased to 2.11 times. Figure 12b shows that in the AAS-LBR scheme, when the initial radius $r_{ini}$ = 30, the broadcast radius of the far-sink node can be adjusted to 3.93 times; when $r_{ini}$ = 60, the broadcast radius of the far-sink node can be adjusted to 1.97 times; when the initial radius $r_{ini}$ = 90, the broadcast radius of the far-sink node can be adjusted to 1.3 times. This is because the farther away from the sink, the more remaining energy there is, and the greater the broadcast after adjusting the broadcast radius with the remaining energy.

**Algorithm 1.** Calculate the Broadcast Radius at a Different Distance from the Sink**Input**: The original broadcast radius $r_{x}\left[k_{0}\right]$, $k_{0}$, the probability of generating data γ and energy parameters in Table 1 and Table 2 **Output**: The radius of the broadcast at distance x
1: Get the transmission power $q_{x}\left[k_{0}\right]$ with $r_{x}\left[k_{0}\right]$ ref. Table 2 2: $e_{max}$
$=e_{x_{0}}\left(k_{0}\right)$; 3: **For** each *x* from $x_{0}$ to R 4:   $k_{cur}=k_{0}$; 5:    Calculate $Q_{r}^{x}$ and $Q_{t}^{x}$ using Equation (9) with $r_{x}\left[k_{0}\right]$ 6:    Calculate $e_{x}$ using Equation (2) with $q_{x}\left[k_{cur}\right]$; 7:    $e_{x}^{\prime }=e_{x}\left(k_{0}\right)+e_{slot}$; 8:   **While**
$e_{max}-e_{x}^{\prime }>0$ 9:     $k_{cur}=k_{cur}+1$; 10:     Get $r_{x}\left[k_{cur}\right]$ according to $q_{x}\left[k_{cur}\right]$ ref. Table 2 11:     Calculating $e_{x}^{\prime \prime }$ using Equation (2) with $q_{x}\left[k_{cur}\right]$ 12:     **If**
$e_{x}^{\prime \prime }$ > $e_{max}$ 13:      $r_{x}\left[k\right]=r_{x}\left[k_{cur}-1\right]$ 14:       break; 15:     **Else if**
$e_{x}^{\prime \prime }$ == $e_{max}$ 16:       The broadcast radius: $r_{x}\left[k\right]=r_{x}\left[k_{cur}\right]$ 17:         break; 18:     **Else if** 19:       $e_{x}^{\prime }$ = $e_{x}^{\prime \prime }$ 20:     **End if** 21:   **End while** 22:   Output: $r_{x}\left[k\right]$, 23: **End For**

Figure 12c shows the energy consumption of the unit data packet transmitted at different distances from the sink calculated by Equation (10). From Figure 12c, it can be seen that when the initial radius $r_{ini}$ = 90, the transmission energy consumption of the far-sink node can be increased to 1.04 times. The above analysis shows that the AAS-LBR scheme can effectively use the remaining energy of the node to adjust the broadcast radius of the node.

#### 4.2.2. Construct Broadcast Backbone

**Figure 13:**
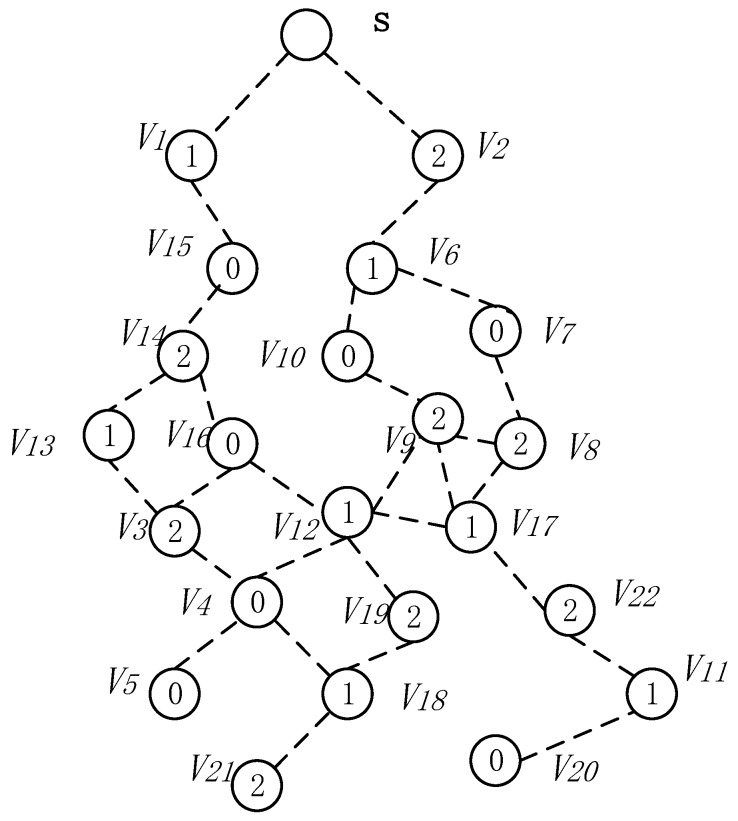
Original physical link.

This section mainly discusses the construct broadcast backbone process. The related concepts used in this process are given in Table 3. Figure 13 is the network diagram used in the process of constructing the broadcast backbone.

**Figure 14:**
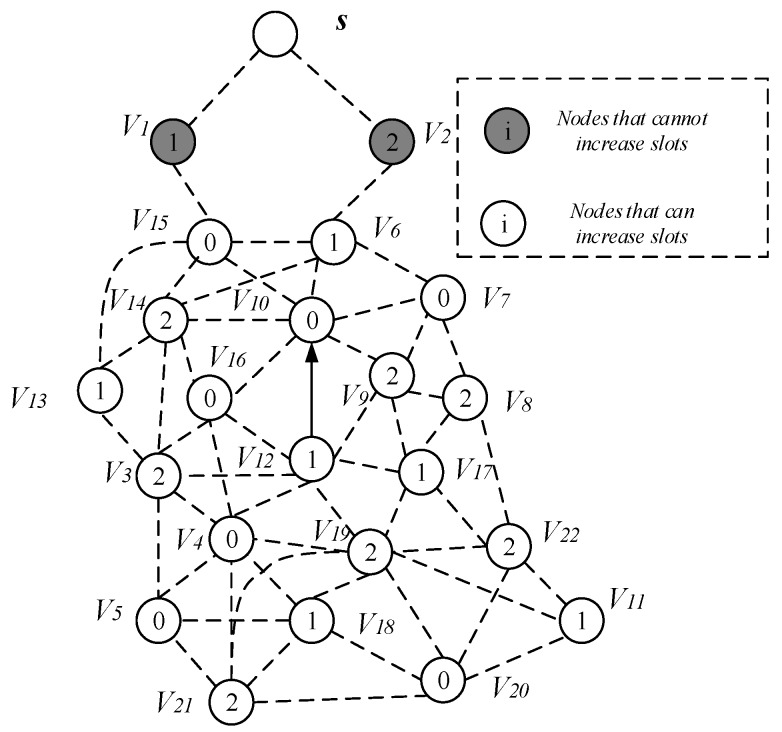
Physical link for AAS-LBR.

The network diagram of Figure 13 shows the links of the node and the active slot of the node selection. Figure 14 shows the network diagram after the calculation of Algorithm 1 to expand the broadcast radius of the node with energy remaining. As the broadcast radius of the node increases, the communication link between the nodes in Figure 14 is increased compared to Figure 13. For example, the node $v_{15}$ in Figure 14 can establish communication with the node $v_{13}$.

Next, we give the process of constructing the broadcast backbone. The process of constructing broadcast backbone can be divided into two steps: (1) finding the Minimum Covering Node Set; (2) constructing the backbone. The specific descriptions are as follows:

(1) Finding the Minimum Covering Node Set

The AAS-LBR scheme in the process of constructing the broadcast backbone, the process of finding the Minimum Covering Node Set is: for each time slot i, each node that is active in the time slot i is grouped into the set $U_{i}$. If the node v can cover the largest number of uncovered nodes in $U_{i}$, then node v is selected as the coverage node of those that are not covered. The node v is merged into the minimum coverage set $C_{i}$, while the node covered by v is marked as the covered node and then deleted from $U_{i}$. Repeat the above process until each node in the set $U_{i}$ is covered by a node, and the resulting set $U_{i}$ = {∅}. All nodes of the covered node know all the information about their covered nodes.

**Figure 15:**
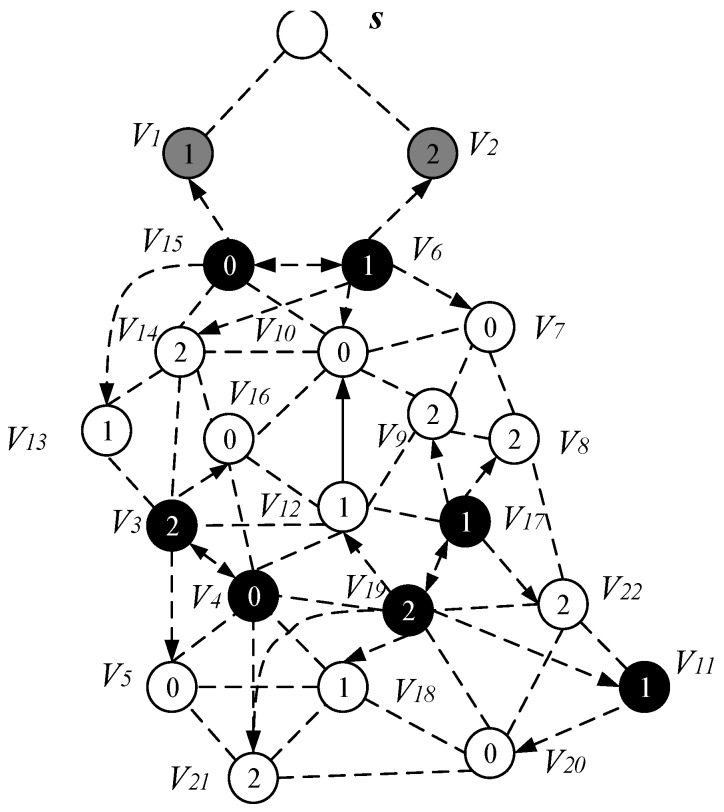
Minimum Covering Node Set.

As shown in Figure 14, for time slot 0, $U_{0}=\left\{v_{4},\;v_{5},\;v_{7},\;v_{10},v_{15},\;v_{16},\;v_{20}\right\}$, $C_{0}$ = {∅} at this time, the node that can cover the most time slot 0 is the node $v_{3}$ and $v_{6}$. Both $v_{3}$ and $v_{6}$ can cover three nodes with time slot 0, which are $v_{4}$, $v_{5}$, $v_{16}$ and $v_{7}$, $v_{10}$, $v_{15}$, respectively. Then we merge $v_{3}$ and $v_{6}$ into the set $C_{0}$, at this time $C_{0}$ = {$v_{3}$, $v_{6}$}, remove the nodes covered by $v_{3}$ and $v_{6}$ from $U_{0}$. There is only one node $v_{20}$ left in $U_{0}$ and the nodes that can cover node $v_{20}$ have $v_{11}$, $v_{18}$, $v_{19}$, $v_{21}$ and $v_{22}$. We choose $v_{11}$ with the smallest id as the covering node of $v_{20}$ (when a node is covered by multiple nodes, we select the node with the smallest id as the covering node), merge $v_{11}$ into the set $C_{0}$, and finally get the Minimum Coverage Set of time slot 0 $C_{0}=\left\{v_{3},\;v_{6},\;v_{11}\right\}$. Then $v_{20}$ is deleted from the set $U_{0}$, and the set $U_{0}=\left\{\emptyset \right\}$ at this time. Next, find the node that can cover the most time slot 1, and repeat the above process until the set $U_{1}$ is also empty. After the above operation, the MNCS of the time slot 1 is $C_{1}$ = {$v_{15}$, $v_{19}$}; similarly, the node that can cover the most time slot 2 is found, and finally the Minimum Coverage Set of the coverage time slot 2 is $C_{2}$ = {$v_{4},v_{6,}v_{17}$}. In this way, all nodes in the network topology diagram can be covered by a node, and the process of finding the Minimum Coverage Set is completed, as shown in Figure 15.

(2) Constructing the backbone

Next is constructing the backbone. The process of constructing the backbone is roughly divided into three steps: (a) build the covering sub-tree; (b) preliminary backbone determination; (c) finalizing the backbone. The following describes the execution process:

(a) Build the covering sub-tree

In building the covering sub-tree, the possible situations and handling are as follows:

*Case 1*. If the covering node v is covered by another u, and the node u is at a higher level than the node *v*, we specify *u* as the parent of v. As in Figure 15, the node $v_{7}$ is covered by the node $v_{6}$, then we specify node $v_{6}$ as the parent of $v_{7}$, $P(v_{7}$) = $v_{6}$.

**Figure 16:**
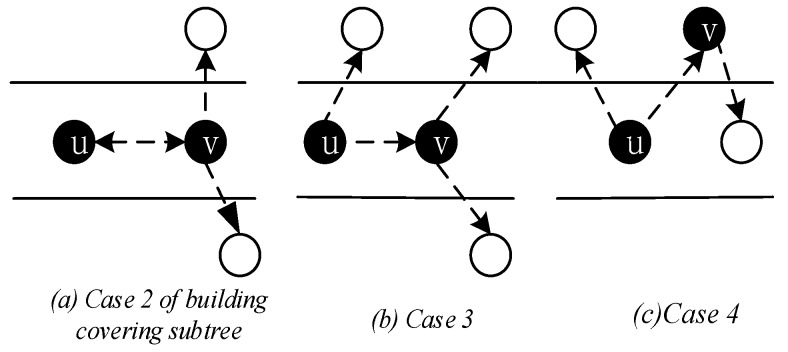
Building covering sub-tree.

*Case 2*. If the two covering nodes *u* and, cover each other and they are at the same level, as seen in Figure 16a, select the node with more neighbors as the parent node of the other node, at this time *P*(*u*) = *v*, *R*(*u*) = *R*(*v*). If the number of neighbors of the two nodes is the same, the parent node with the smallest node id is selected. For example, Figure 15, the two covering nodes $v_{15}$ and $v_{6}$, cover each other and they are at the same level, $v_{6}$ and $v_{15}$ have the same number of neighbor nodes. We choose $v_{6}$ with small id as the parent node of $v_{15}$, so here *P*($v_{15}$) = $v_{6}$, *R*($v_{6}$) = *R*($v_{15}$).

*Case 3*. If two covering nodes, *u* and v are at the same level and *u* covers v, like in Figure 16c, then *P*(*v*) = *u* and no cycle shall be generated. If *R*(*u*) is not set yet, *u* itself is selected as the root node. And R(u)=R(v). As shown in Figure 16b, *P*(*v*) = *u*, if R(u)=∅, then R(u)=u,R(v)=R(u)=u.

*Case 4*. If the covering node v does not satisfy any of the above, it is added to the subtree set by only one node, then this is the default case and v is selected as the root node. As shown in Figure 16c, P(v)=∅, R(v)=v.

(b) Preliminary backbone determination

**Figure 17:**
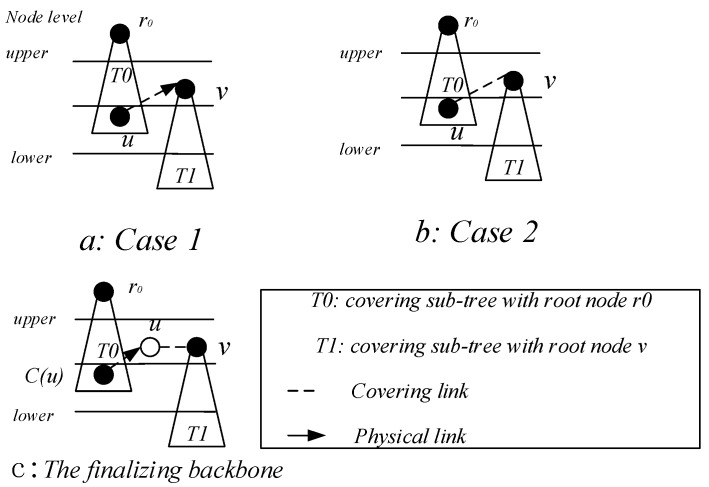
Case 1 and case 2 in finalizing backbone.

Through the situation described above, from low-level to high-level traversal, there are three situations and handling for covering nodes that still do not have a parent node:

*Case 1*. Node v can find a covering node u at a lower level, and u can cover v. In the build the covering sub-tree phase, only the lower level covering nodes are connected to the same level of covering nodes or higher level. There is a case where a covering sub-tree has a root node v, and the root node v is at a higher level with respect to its covering node u, but at a lower level than the root node of u, at this time $P\left(v\right)=u,R\left(v\right)=R\left(u\right)=r_{0}$, as shown in Figure 17a.

*Case 2*. If node v cannot find the parent node in Case 1. We try to find the node u, u must satisfy one of the following two situations: (i) node u is the neighbor node of node v, node u already exists in covering sub-tree, and the root node of u is at a higher level than node v, at this time P(v)=u,R(v)=R(u), as shown in Figure 17b; (ii) u is a connection point, the root node of C(u) is higher than node v, at this time, u is added to the subtree, P(v)=u, P(u)=P(C(u)), R(v)=R(u)=R(C(u)), as shown in Figure 17c.

**Figure 18:**
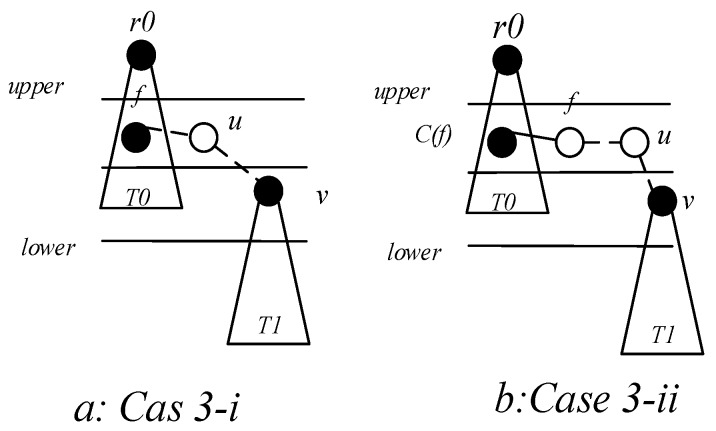
Connection T1 to T0 using one-hop and two-hop forwarders.

**Figure 19:**
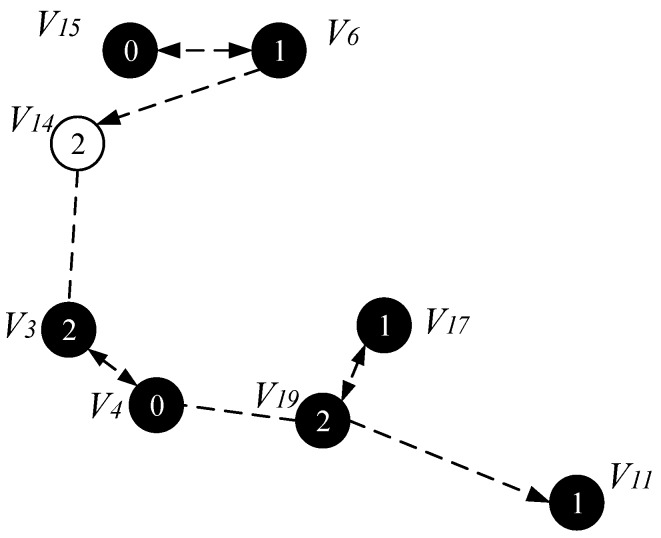
Preliminary broadcast backbone of AAS-LBR scheme.

*Case 3*. If node v cannot find the parent node through case 1 and case 2, then find node u and f which must satisfy one of the following two conditions: (i) u is a connection point, f is already present in sub-tree and u is the neighbor node of f. The root node R(f) of f is at a higher level than node v, at this time P(u)=f, P(v)=u, R(v)=R(f), as shown in Figure 18a; (ii) u is a connection point, f is another connection point, u is the neighbor node of f, and the root node of C(f) is at a higher level than node *v*, at this time, P(f)=C(f), P(u)=f, P(v)=u, R(v)=R(u)=R(f)=R(C(f)), as shown in Figure 18b. The resulting preliminary backbone is shown in Figure 19.

(c) Final backbone

In the process of constructing the final backbone, firstly, we start from the root node of the preliminary backbone obtained from the above operation, traverse from the upper to the low level, adding a time slot to the node satisfying the time slot addition condition.

**Figure 20:**
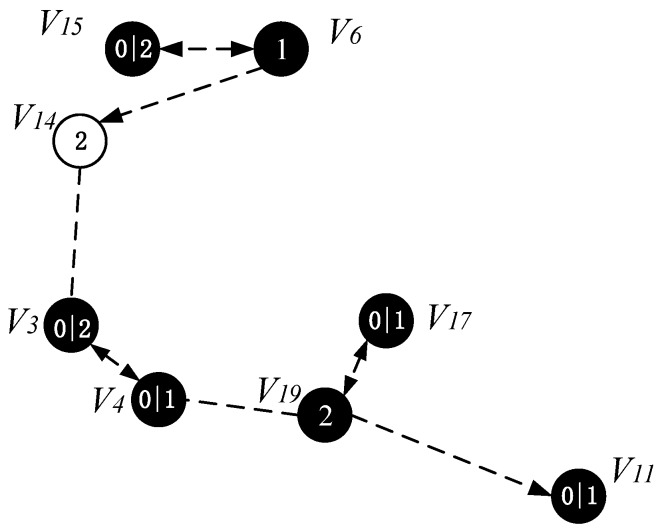
Broadcast backbone of AAS-LBR scheme.

The specific operation of adding an active slot is as follows: if the time slot of the node is the next time slot of the active node of its parent node, there is no need to increase the time slot temporarily; instead, we add a time slot for the node to the next time slot of the active slot of its parent node. As shown in Figure 20, the parent node of $v_{15}$ is $v_{6}$, $v_{15}$ satisfies the condition of adding one time slot, then we add a time slot 2 for $v_{15}$ and the time slot of $v_{14}$ is exactly the next time slot of $v_{6}$ active slot, so the time slot of $v_{14}$ does not need to be temporarily added. Repeating the above operation, $v_{3}$ adds a time slot 0, $v_{4}$ adds one time slot 1, $v_{11}$ and $v_{17}$ add one time slot 0. The resulting backbone is shown in Figure 20.

The list of time slots for receiving and transmitting data for each node of the AAS-LBR scheme is shown in Table 4 below. 

In order to compare the proposed AAS-LBR scheme strategy with the previous strategy, we also give the broadcast process for the network of Figure 13 under the ABRCD strategy [17].

**Figure 21:**
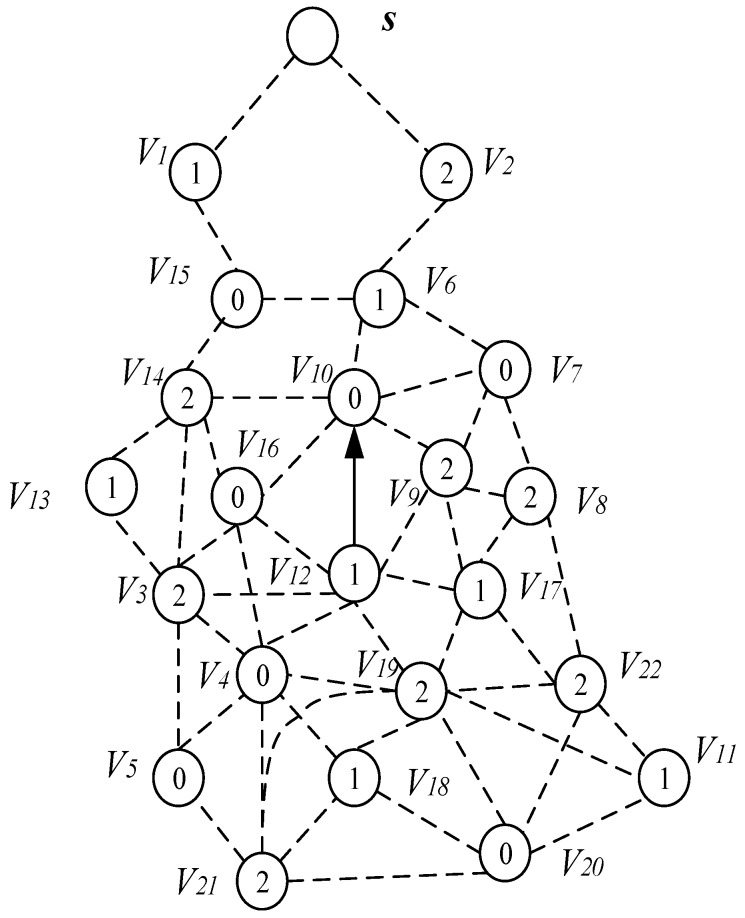
Physical link for ABRCD.

**Figure 22:**
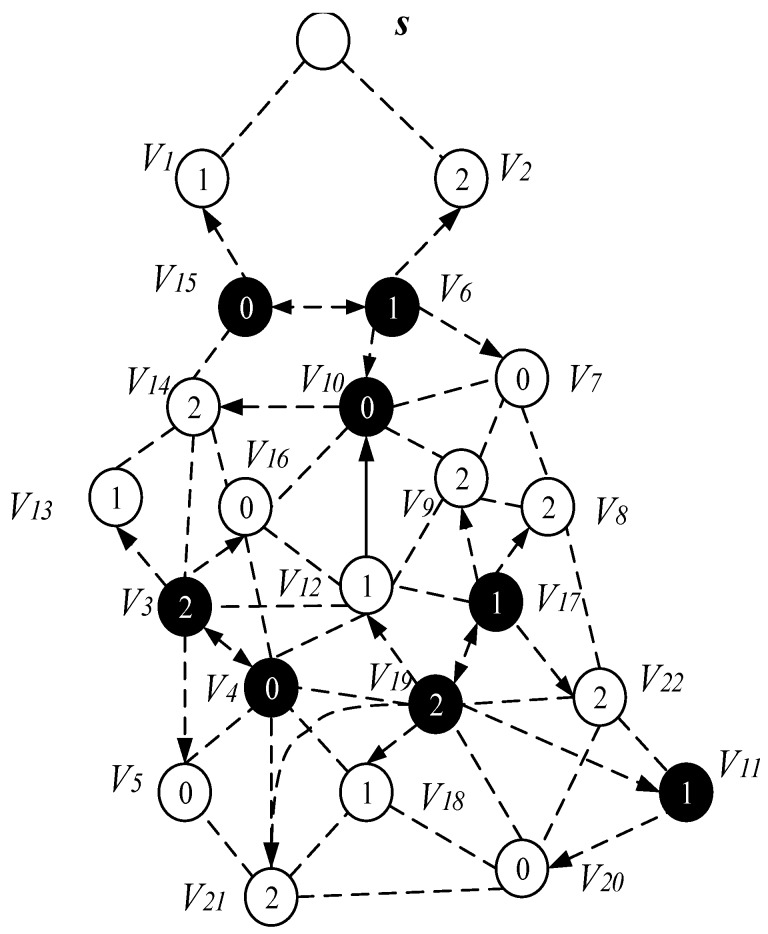
Minimum covering node set.

**Figure 23:**
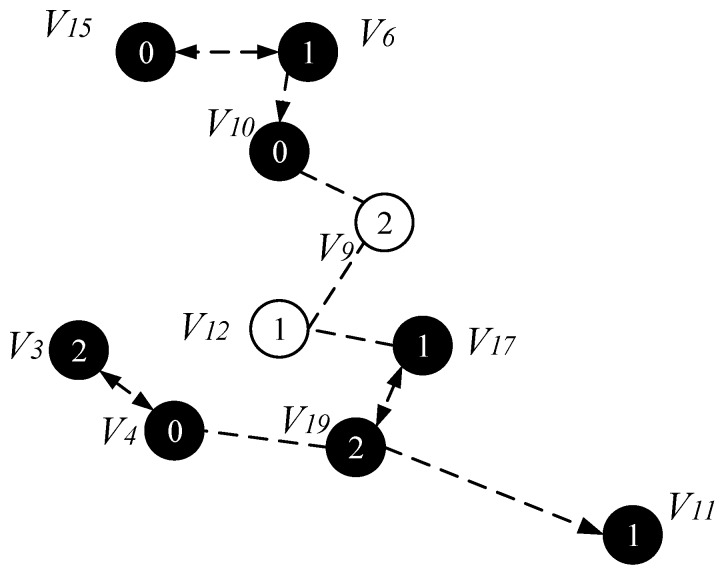
Broadcast backbone for ABRCD.

Figure 21, Figure 22 and Figure 23 shows the process of constructing a broadcast backbone by ABRCD. Figure 21 shows the physical link diagram obtained by the ABRCD scheme using a proportional increase in the broadcast radius. The minimum coverage set of nodes obtained by the ABRCD scheme in the first stage of constructing the broadcast backbone is shown in Figure 22. The final backbone obtained by the ABRCD scheme is shown in Figure 23.

The list of time slots for receiving and transmitting data for each node in the ABRCD scheme is given in Table 5 below.

It can be seen from the above experimental results: the number of broadcasts of the AAS-LBR scheme is eight, and the delay is six. In the ABRCD scheme, when data is transmitted from sink to each node, its broadcast number is 10 and the delay is 11. Compared with the ABRCD scheme, the AAS-LBR scheme reduces the number of broadcasts by 20% and the broadcast delay by 45.5%. Obviously, adjusting the duty cycle and the broadcast radius by the remaining energy of the node can effectively reduce the number of broadcasts and broadcast delay.

#### 4.2.3. Code Dissemination

Since MCNS has covered the entire network, therefore, after the nodes on the broadcast backbone get the codes, the nodes in the MCNS only need to broadcast the code to the nodes covered by it. In the AAS-LBR scheme, unlike the previous strategy, since the active of the node can be increased, the active slot of the covered node is selected to be exactly the same as the time slot of the dominant node. Thus, in MCNS, when the broadcast backbone is completed, the node that can increase the active slot has already obtained the code. The specific method of adjusting the active slot of the node is as follows: Traverse the final backbone from top to bottom, the set P is all the nodes that satisfy the addition of one time slot. The node v is the node in the set P, the active slot of v is the slot α, and the node u is the covering node of v. Then v selects one of the time slots of u as the time slot to be increased, and the time slot satisfies the following conditions: (i) the time slot of the node v is different from the active time slots *β* of the u, i.e., α≠β, then the node v adds a time slot *β*; (ii) the time slot of node v is different from all time slots of u, and the time slot added by node v is the smallest time slot of u. After the above operation, node v is deleted from set P. The above operation is repeated until the set P is an empty set, such all nodes that can increase the time slot have added one time slot. Algorithm 2 is described as follows:

**Algorithm 2.** Adjust the Duty Cycle of the NodeInput: *G* = (*V*,*E*), *A*(*v*),∀ *v*∈
*V*, and *P* 1:   **While**
*P*
≠∅
**Do** 2:       Find the node *u* that covering *v* 3:       **For** the time slot of *u* 4:          **If** the time slot β of *u*, β∉
*A*(*v*) 5:             Node *v* adds a time slot: *A*(*v*) = *A*(*v*)∪β 6:          **Else**
7:             Node *v* adds a time slot: *A*(*v*) = *A*(*v*)∪β, 8:         β is the smallest time slot in *u* 9:        **End for** 10:       Remove node *v* from collection *P*: *P* = *P*\*v* 11:    **End while**

**Figure 24:**
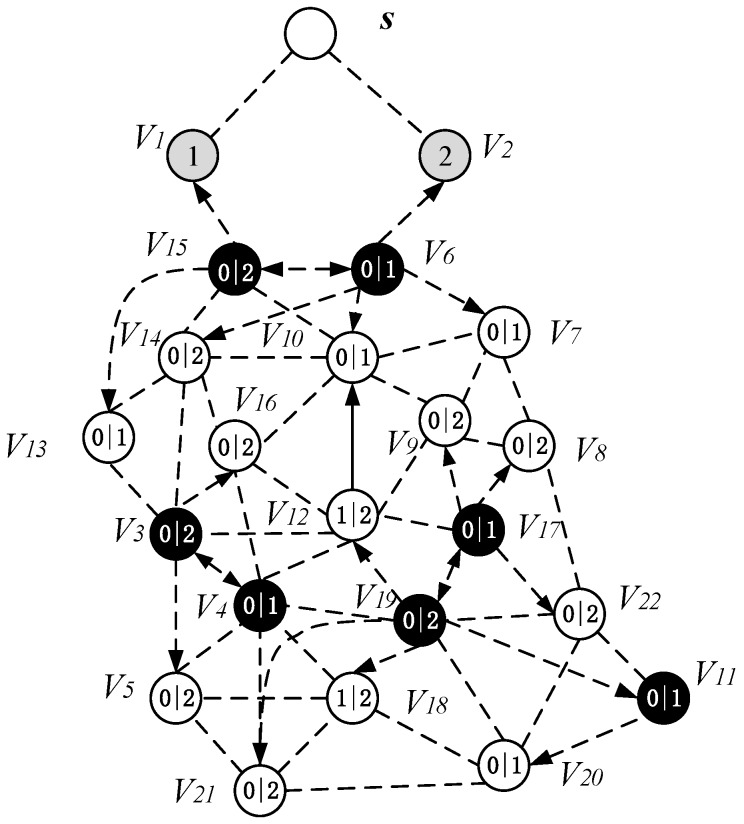
Topology diagram after adjusting node time slot.

It is worth noting that some nodes in the backbone do also not exist in the set P except that the nodes in the near-sink area do not satisfy the condition of increasing the duty cycle. This is because these nodes have been added time slot in the process of construct broadcast backbone. The topology of the node time slot is adjusted as shown in Figure 24.

## 5. Theoretical Analysis

### 5.1. Analysis of Energy Consumption

In this paper, we use Equation (1) to calculate the energy consumption of the node. The value of the corresponding parameter comes from Table 1.

**Figure 25:**
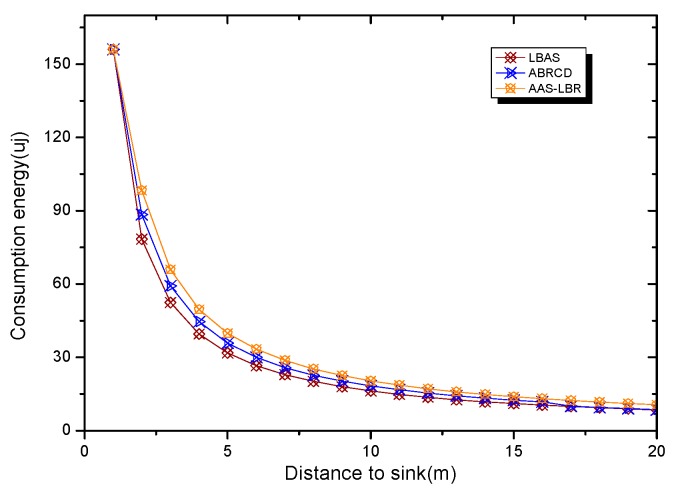
Energy consumption of nodes 1–20 m away from sink under T=20 and r=28 m.

**Figure 26:**
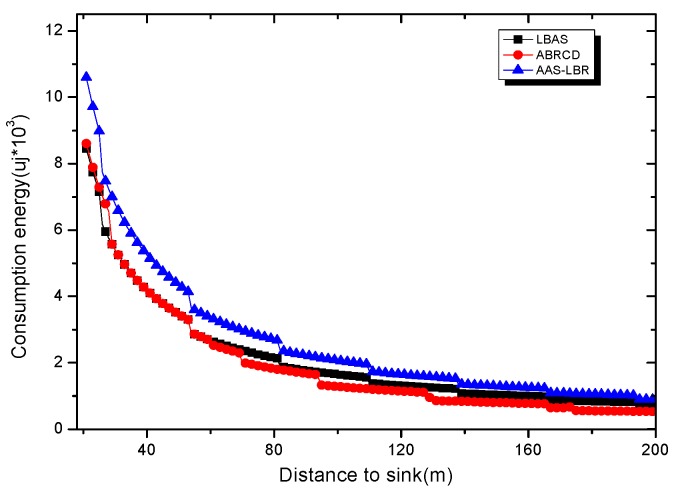
Energy consumption of nodes 20–200 m away from sink under T=20 and r=28 m.

**Figure 27:**
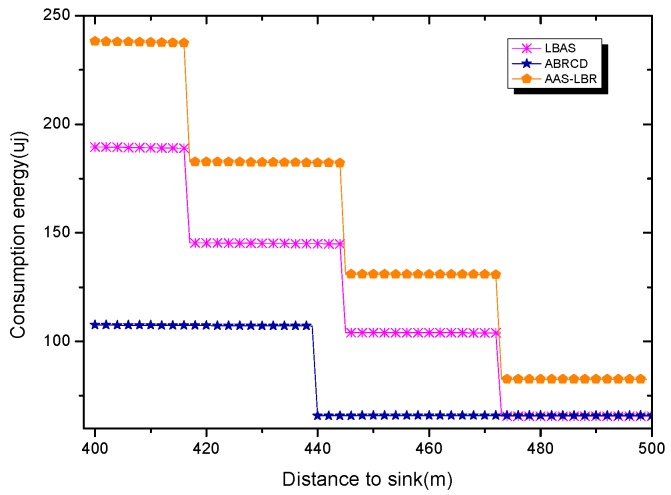
Energy consumption of nodes 400–500 m away from sink under T=20 and r=28 m.

**Figure 28:**
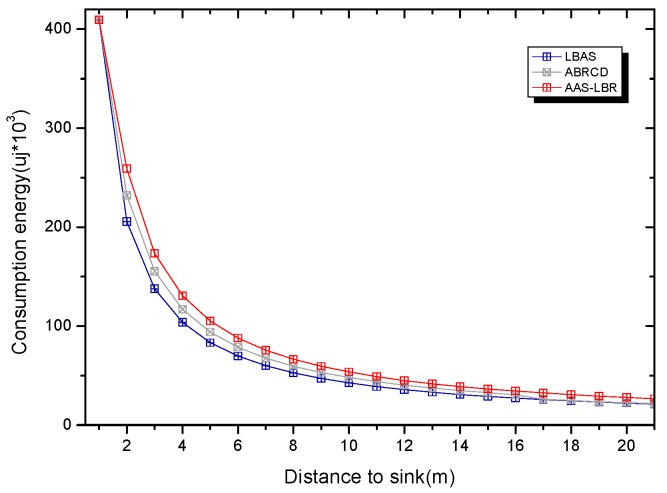
Energy consumption of nodes 1–20 m away from sink under T=60 and r=28 m.

**Figure 29:**
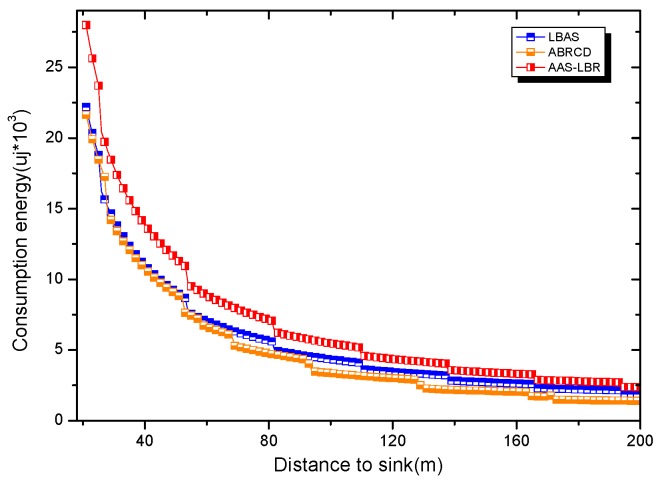
Energy consumption of nodes 20–200 m away from sink under T=60 and r=28 m.

**Figure 30:**
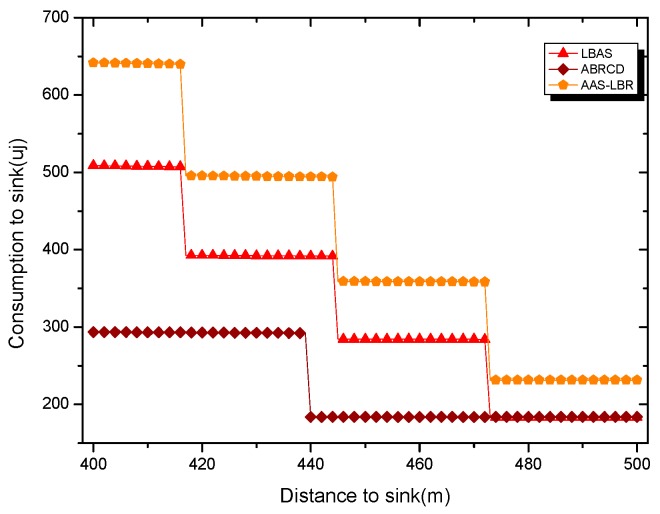
Energy consumption of nodes 400–500 m away from sink under T=60 and r=28 m.

**Figure 31:**
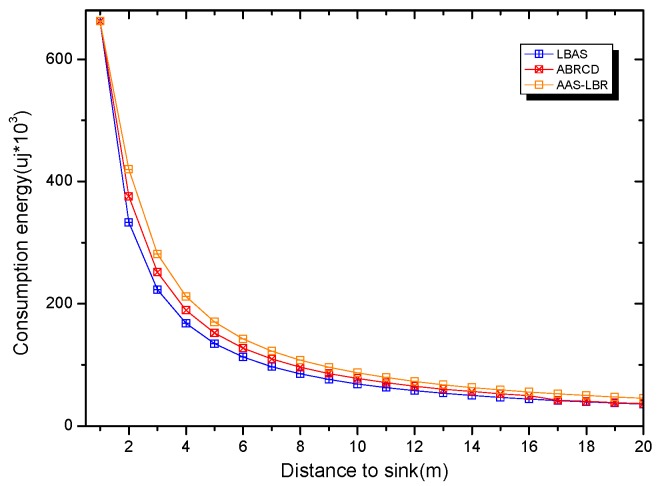
Energy consumption of nodes 1–20 m away from sink under T=100 and r=28 m.

**Figure 32:**
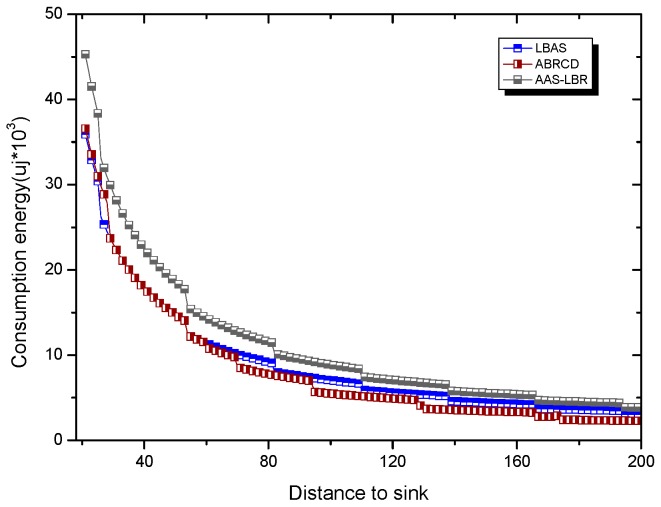
Energy consumption of nodes 20–200 m away from sink under T=100 and r=28 m.

**Figure 33:**
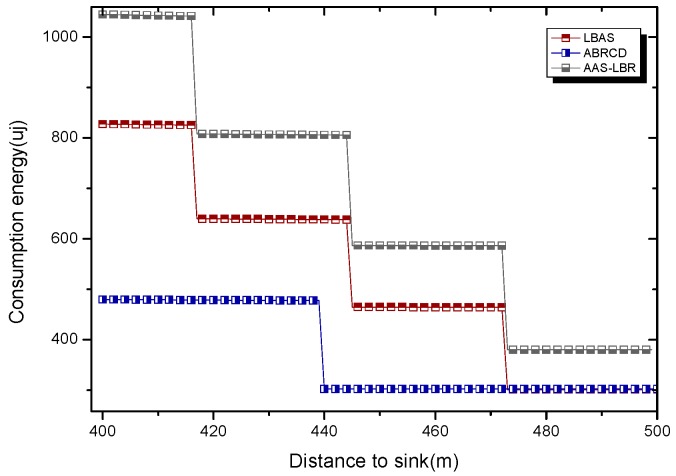
Energy consumption of nodes 400–500 m away from sink under T=100 and r=28 m.

Under the parameter values $R=500,\;\gamma =0.1,\;E_{0}=0.1j,\;r=28,$ the energy consumption $e^{x}$ at the distance sink xm is analyzed for the period T=20, T=60 and T=100. Figure 25, Figure 26 and Figure 27 show that the energy consumption of the node far from sink xm is analyzed in the case of the initial broadcast radius r=28 m and period T=20, that is, the effect of adjusting the duty cycle and the broadcast radius of the node on the energy consumption of the node; Figure 25 represents the energy consumption of the node at 1–20 m from the sink node; Figure 26 represents the energy consumption of the node at the distance of 20–200 m from the sink node and Figure 27 indicates the energy consumption of the node at a distance of 400–500 m from the sink node; Figure 28, Figure 29 and Figure 30 show the energy consumption of the nodes at different distances from the sink in the case of the initial broadcast radius r=28 and the period T=60. Figure 31, Figure 32 and Figure 33 show the effect of the three schemes on the node energy consumption at different distances from the sink in the case of the initial broadcast radius r=28 and the period T=100.

As can be seen from Figure 25, Figure 26, Figure 27, Figure 28, Figure 29, Figure 30, Figure 31, Figure 32 and Figure 33, the energy consumption of the nodes under the three schemes: (1) the energy consumption of the node near the sink is higher than the energy consumption of the node away from the sink; (2) the maximum energy consumption of the nodes in the AAS-LBR scheme is the same as the maximum energy consumption in the other two schemes; (3) the energy consumption of the nodes in the non-hotspot area in the AAS-LBR scheme is higher than the energy consumption of the nodes in the non-hotspot areas in the other two schemes. The reason is that the far sink node in the AAS-LBR scheme consumes energy by adjusting the duty cycle and the broadcast radius without affecting the network lifetime. If there is enough remaining energy of the far-sink area, the node can not only add an active time slot but also adjust the broadcast radius of the node under AAS-LBR.

From Figure 25, Figure 28 and Figure 31, it can be seen that the energy consumption of the nodes under the three schemes when T takes different values: (1) the energy consumption in the near-sink region increases with the value of T, and the corresponding energy consumption increases; (2) the maximum energy consumption in the AAS-LBR scheme is the same as the maximum energy consumption in the other two schemes; (3) the energy consumption of the nodes in the non-hotspot area in the AAS-LBR scheme is higher than the energy consumption of the nodes in the non-hotspot areas in the other two schemes. The reason is that the value of T increases, which leads to an increase in the energy consumption of the near sink node and a relative increase in the remaining energy of the far-sink area. After the broadcast radius is expanded by using the remaining energy, the transmission power of the node is increased, and the corresponding energy consumption is also increased. 

From Figure 27, Figure 30 and Figure 33, it can be seen that under the three schemes, the energy consumption at a distance of 400–500 m is obtained. Obviously, because the remaining energy of the far-sink node in the AAS-LBR scheme is used to adjust the duty cycle and the broadcast radius, the energy consumption away from the sink node is more than the other two schemes.

### 5.2. Analysis of Energy Utilization Ratio and Network Lifetime

**Figure 34:**
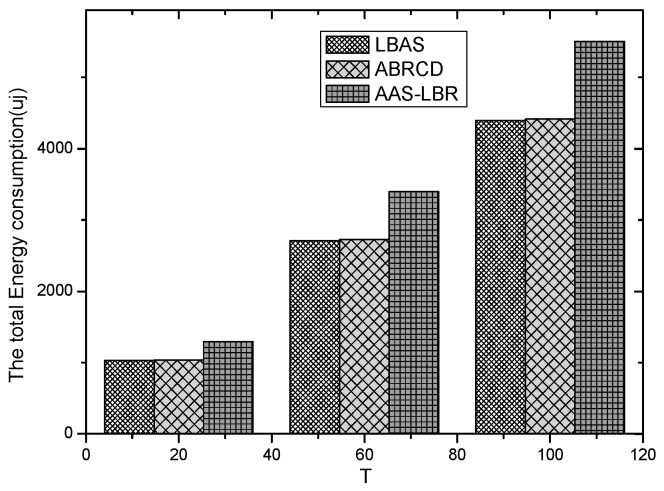
Total energy consumption of three schemes under different cycles with *r* = 28 m.

As can be seen from Figure 34, the total energy consumption of the entire network in the AAS-LBR scheme is higher than the total energy consumption of the other two schemes. The reason is that compared to the other two schemes, if there is enough remaining energy in the far-sink node area of the AAS-LBR scheme, it will be used to adjust the duty cycle and the broadcast radius. As a result, the energy consumption of the node in the far sink area increases, resulting in an increase in the energy consumption of the overall network.

**Figure 35:**
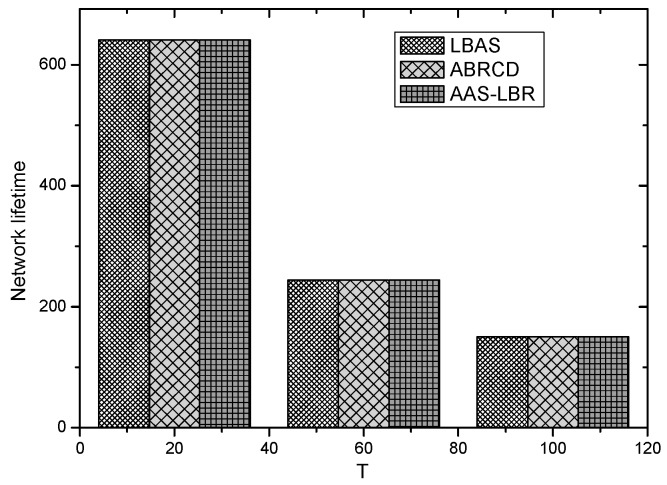
Network lifetime of three schemes under different cycles with *r* = 28 m.

The node lifetimes of the three schemes can be calculated according to Equation (5). In the network with r=28 m, the influence of T on different values can be seen from Figure 35. In a network with r=28 m, the lifetime of the three schemes is the same when the initial energy $E_{0}$ of the nodes is the same. The reason is that the energy consumption of the nodes in the near-sink area of the three schemes is the same, resulting in the same network life under the three schemes. It shows that the AAS-LBR scheme can make the energy consumption more balanced without affecting the network life.

**Figure 36:**
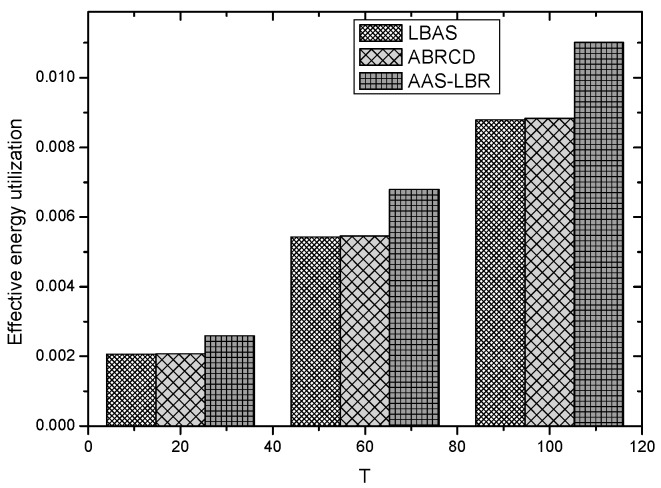
Effective energy utilization of three schemes under different cycles with *r* = 28 m.

The energy utilization rate of the three schemes can be calculated according to Equation (6). In the network with r=28 m, the influence of the value of different T on the energy utilization can be seen from Figure 36. It can be seen from Figure 36 that the energy utilization rate of the AAS-LBR scheme is higher than that of other schemes regardless of the value of T. Because the remaining energy of the far-sink node is used to adjust the duty cycle and the broadcast radius of the node without affecting the overall life of the network, the energy consumption of the node is increased, and the overall energy consumption of the network is increased. The utilization rate has naturally increased.

It can be seen from Figure 34, Figure 35 and Figure 36 that the AAS-LBR scheme uses the remaining energy of the node to adjust the duty cycle and the broadcast radius of the node without affecting the overall network life, which is superior to improving the energy utilization.

## 6. Experimental Results Analysis of ABRCD Scheme

The simulation is performed in the Matlab language environment. Matlab is a high-level language for algorithm development, data visualization, data analysis and numerical calculation. The constants used in the experiment are as follows: the radius of the whole network is R = 500 m, the probability of data generation is γ = 0.1 and the initial broadcast radius of the node is r=28.

### 6.1. Transmissions Analysis

**Figure 37:**
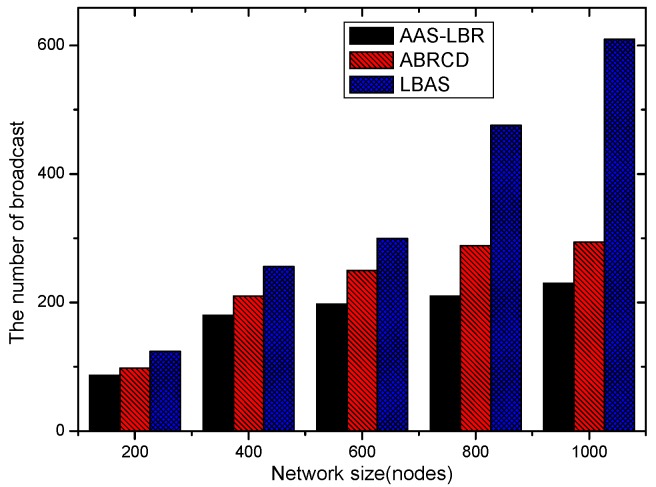
The impact of network size with fixed |*T*| = 30 on the number of broadcasts.

**Figure 38:**
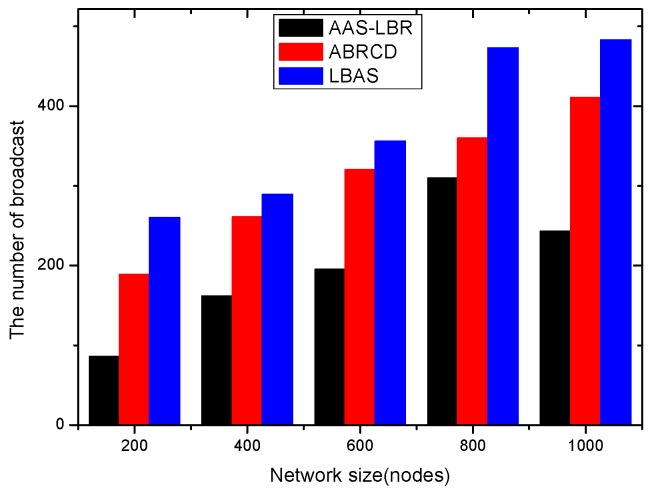
The impact of network size with fixed |T| = 90 on the number of broadcasts.

The number of broadcasts at |T|=30 and |T|=90 can be seen in Figure 37 and Figure 38, respectively. The number of broadcasts in the AAS-LBR scheme is smaller than the number of broadcasts in the other two schemes. The number of broadcasts usually increases as the size of the network (i.e., the number of nodes) increases. Obviously, in the case of |T| predetermined, when the number of nodes is increased, the program code can be selected to cover nodes at different duty ratios when diffused to all nodes in the network, which leads to an increase in the number of broadcasts. 

**Figure 39:**
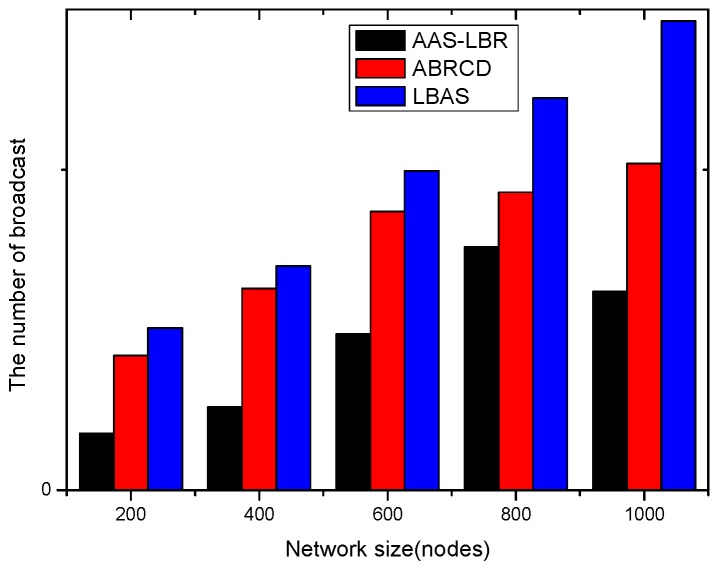
The impact of network size with fixed |T|= 150 on the number of broadcasts.

**Figure 40:**
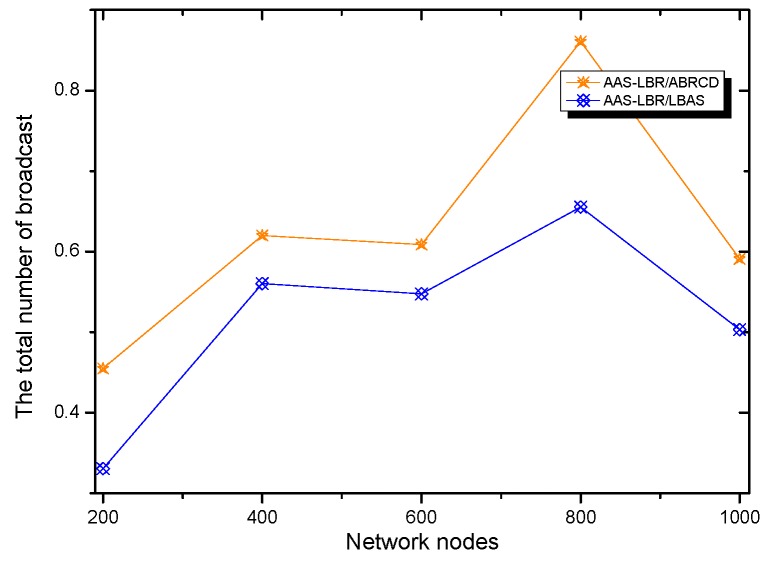
The impact of network size with fixed value of |T|= 90 on the total number of broadcast.

The number of broadcasts at |T|=150 is given in Figure 39. It can be seen that the number of broadcasts in the AAS-LBR scheme is less than the number of broadcasts of the other two schemes. The reason is that when the program code is spread from the sink node along the broadcast backbone to all nodes, the number of broadcasts is affected by the number of neighbor nodes and time slots. When there are few neighbor nodes, the code is propagated to all nodes, and multiple hops are required to be broadcast. When the active slots of the neighbor nodes of the node are different, the node needs to broadcast multiple times to broadcast the code to all neighbor nodes. In the AAS-LBR scheme, the duty cycle and the broadcast radius of the node are increased, the number of nodes establishing communication with the node is increased, the activity time of the neighbor node of the node is increased, and the activity time is earlier. Therefore, a node only needs to broadcast once to broadcast program code to all neighbor nodes to reduce the number of broadcasts. The number of broadcasts in the AAS-LBR scheme improved by 44.51–86.18% compared to LBAS (see Figure 40).

**Figure 41:**
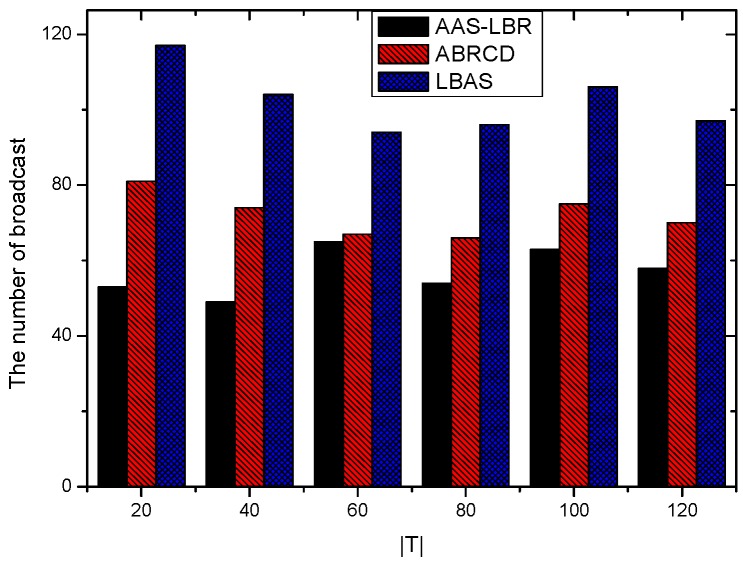
The impact of |T| with fixed network nodes = 200 with fixed on the total number of broadcast.

**Figure 42:**
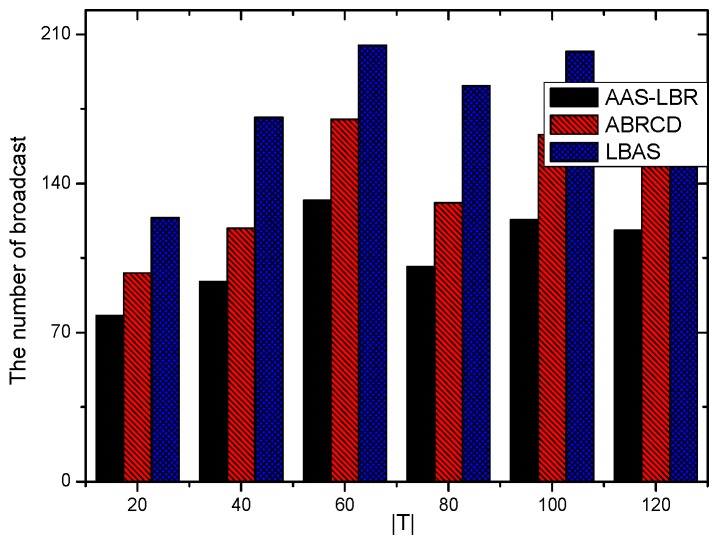
The impact of |T| with fixed network nodes = 400 with fixed on the total number of broadcast.

**Figure 43:**
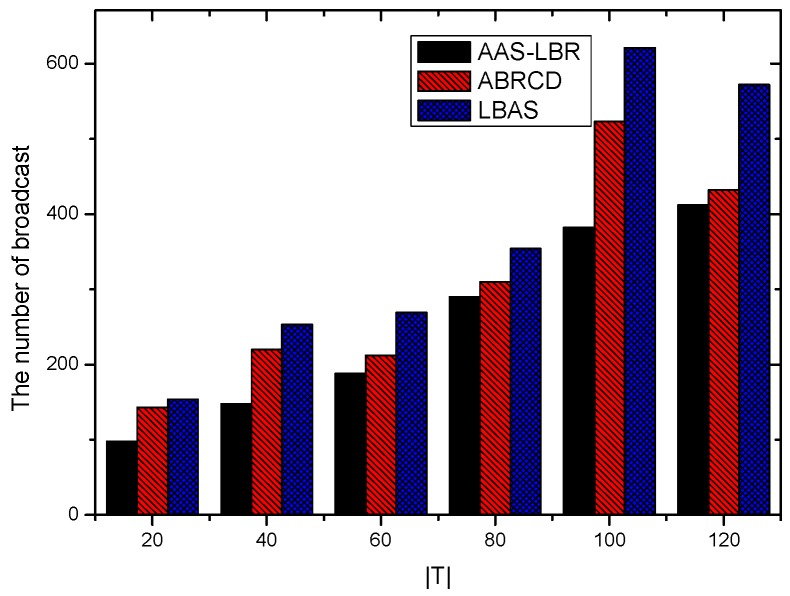
The impact of |T| with fixed network nodes = 600 with fixed on the total number of broadcast.

The total number of broadcasts at network nodes 200 and 400 is shown in Figure 41 and Figure 42, respectively. Regardless of the number of nodes is 200 or 400, the number of broadcasts in the AAS-LBR scheme is smaller than the other two schemes. In Figure 42, when |T|=20, the number of broadcasts in the AAS-LBR scheme is 0.9701 times in the ABRCD scheme; when |T|=40, the number of broadcasts in the AAS-LBR scheme is 0.6443 times that of the ABRCD scheme. The number of broadcasts when the number of network nodes is 600 is given in Figure 43. It can be seen from the figure that the number of broadcasts in the AAS-LBR scheme is still smaller than the other two schemes.

The above experiments show that the AAS-LBR scheme uses the remaining energy of the node to adjust the duty cycle and the broadcast radius, which can effectively reduce the number of broadcasts, and the AAS-LBR scheme has better performance than the other two schemes.

### 6.2. Delay Analysis

**Figure 44:**
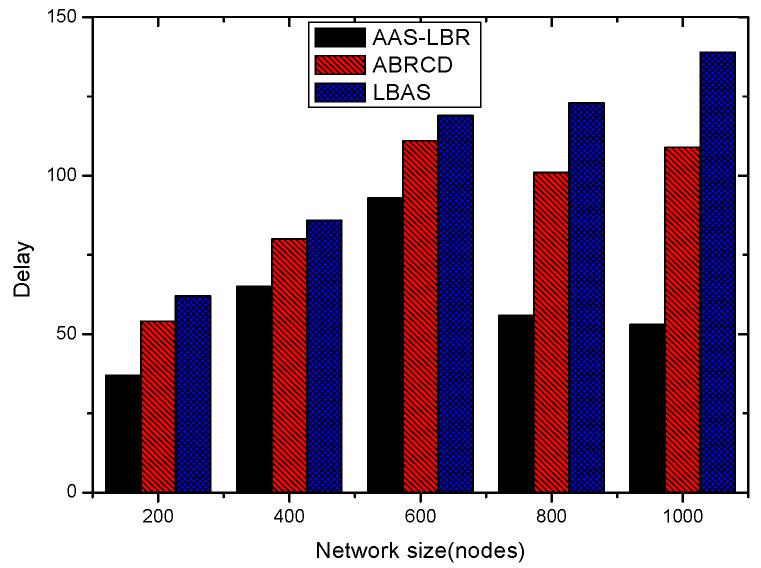
The impact of network size with fixed value of |*T*| = 30 on the broadcast delay.

**Figure 45:**
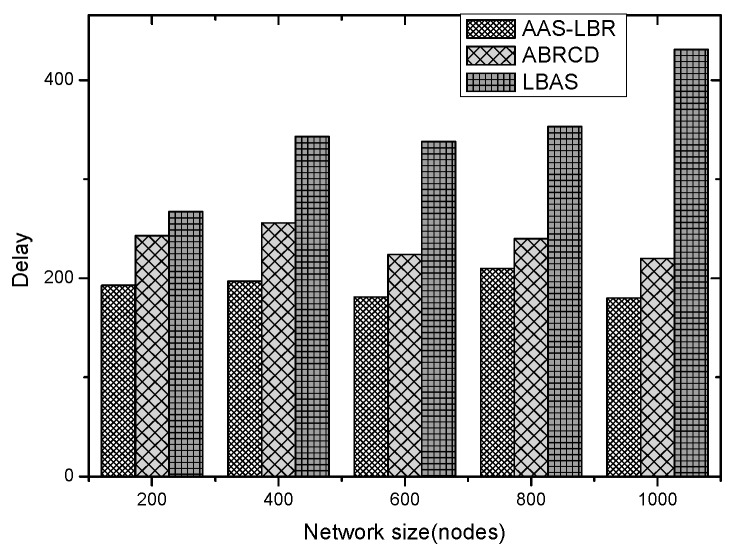
The impact of network size with fixed value of |*T*| = 90 on the broadcast delay.

**Figure 46:**
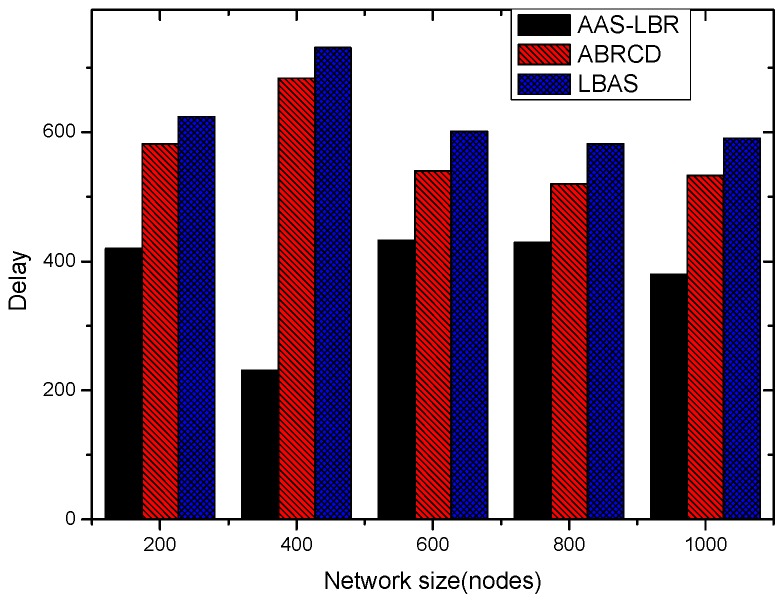
The impact of network size with fixed value of |*T*| = 150 on the broadcast delay.

The broadcast delays at |T|=30 and |T|=90 are shown in Figure 44 and Figure 45, respectively. The broadcast delay in the AAS-LBR scheme is smaller than the other two schemes. The effect of network size (i.e., number of nodes) on the broadcast delay in a network with |T|=150 is shown in Figure 46. It can be seen that the delay of the AAS-LBR scheme is less than the delay of the other two schemes. Obviously, as the size of the network increases, the delay usually increases. Obviously, as the network scale increases, the delay usually increases. Because in the case of |T| predetermined, when the number of nodes is increased, the program code is extended to all nodes in the network, and nodes can be selected as coverage nodes under multiple duty cycles, and the corresponding delay is also increased.

**Figure 47:**
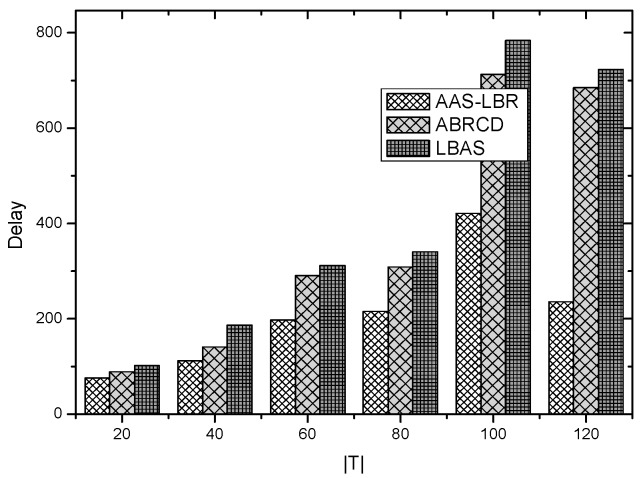
The impact of |*T*| with fixed network nodes = 400 with fixed on the delay.

**Figure 48:**
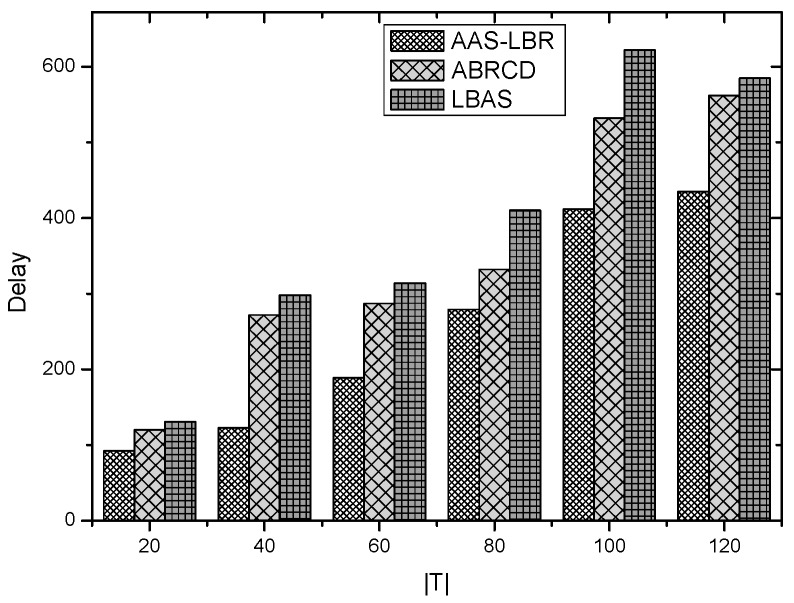
The impact of |*T*| with fixed network nodes = 600 with fixed on the delay.

**Figure 49:**
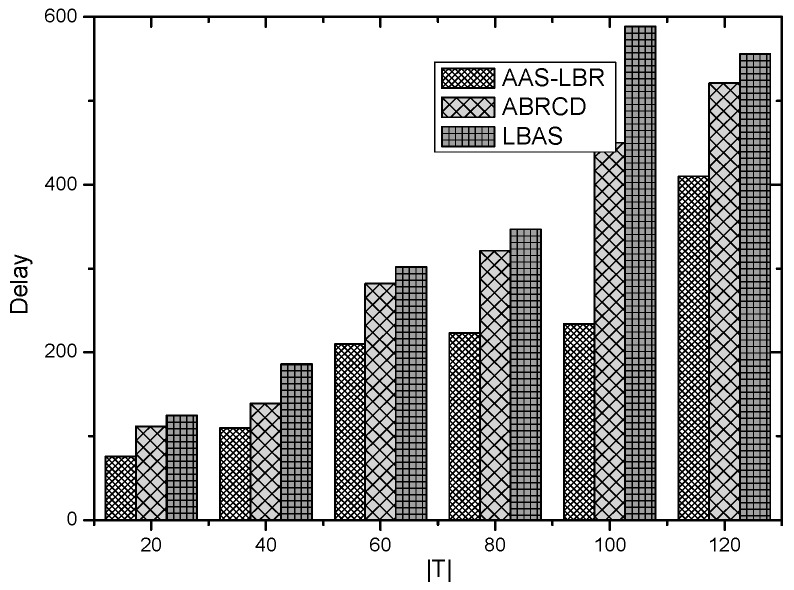
The impact of |*T*| with fixed network nodes = 900 with fixed on the delay.

**Figure 50:**
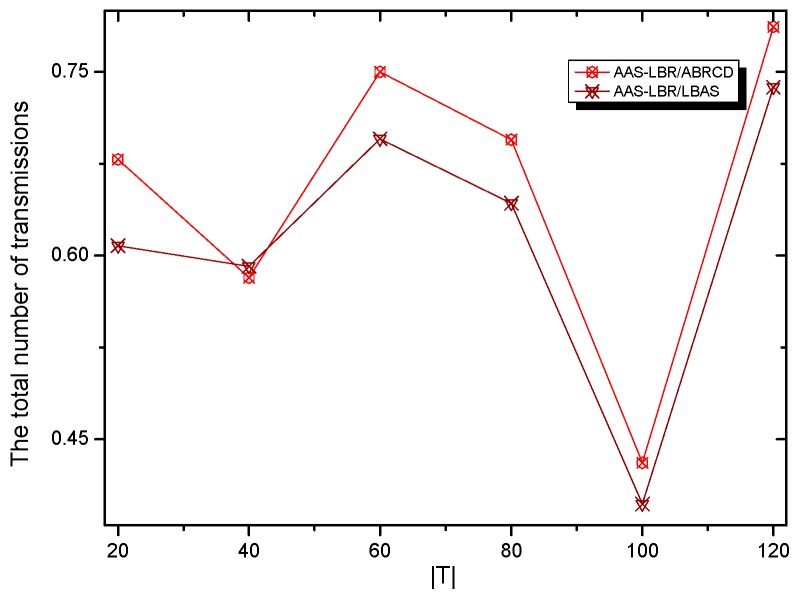
The impact of |*T*| with fixed network nodes = 800 with fixed on the total number of transmissions.

In networks with 400 and 600 nodes, the propagation delay varies with the value of |T| in Figure 47 and Figure 48. It can be seen that the delay in the AAS-LBR scheme is smaller than the others two schemes. The reason is that in the other two schemes, in order to achieve the purpose of reducing the number of broadcasts, it is necessary to establish a broadcast tree. The first stage of establishing a broadcast tree is to find the MSCN. The broadcast backbone contains all the coverage nodes found in the first stage. The coverage nodes have many neighbor nodes, and these neighbor nodes have independent different active time slots. Therefore, when the program code is spread from the sink node along the broadcast backbone to all nodes, due to the different active slot of the neighbor node, one node needs to broadcast multiple times to transmit data to the neighbor node. In the AAS-LBR scheme, the duty cycle and broadcast radius of the node increase, the number of nodes that establish communication with the node increases, the active time of the neighbor nodes of the node increases, and the active time is earlier. Such a node only needs to broadcast once to broadcast the program code to all neighbor nodes, resulting in a decrease in delay. The broadcast delay in a network with 900 nodes is shown in Figure 49. It can be seen that the delay of the AAS-LBR scheme is smaller than the delay of the ABRCD scheme. The reason is the same as above. The node only needs to broadcast once to transmit the program code to all neighbor nodes, thereby reducing the broadcast delay. The program code transmission delay of AAS-LBR is reduced by 43.09–78.69% compared to LBAS (see Figure 50).

The above experiments show that the AAS-LBR scheme uses the residual energy of the node to adjust the duty cycle and the broadcast radius, which can effectively reduce the delay, and the AAS-LBR scheme has better performance than the other two schemes.

## 7. Summary and Conclusions

In this section, we first summarize our work. The main innovation proposed is adding an Active Slot joint Larger Broadcast Radius (AAS-LBR) scheme for reducing the number of broadcast and fast code dissemination while retaining higher network lifetime for WSNs. The AAS-LBR scheme has several innovations: (a) The increased active slot and broadcast radius is increased by using the remaining energy of the far-sink area node, so the AAS-LBR scheme does not reduce the network lifetime compared to other strategies. (b) We confirmed that adding only one active slot would effectively reduce the delay of code dissemination. First, in the AAS-LBR scheme, the active slots are added with a pipeline style for the nodes in broadcast backbone, which enables the code dissemination process to continue without the pause, so the code dissemination delay in the broadcast backbone can be greatly reduced. Second, for the nodes in non-broadcast backbone, the active slots are added coincident with their dominator, so when all the nodes on the broadcast backbone get the code, all the nodes in the network get the code, which further reduces the delay. (c) Combining the broadcast radius of the node with the remaining energy area with the increase of the active slot further reduces the delay. (d) Through theoretical and experimental analysis, the performance of the AAS-LBR scheme greatly optimizes all known scheme. In the AAS-LBR scheme, the transmission delay can be reduced 43.09–78.69% (see Figure 50), the number of broadcasts can be reduced 44.51–86.18% (see Figure 40) and the energy efficiency is improved by about 24.5% (see Figure 36). Table 6 below is a comparison of the key performance delays and broadcast times of AAS-LBR and some proposed effective strategies. It can be seen from Table 6 that AAS-LBR has a great improvement in key performance. Table 7 is a comparison of energy consumption of different strategies. It can be seen that the AAS-LBR strategy also has better performance in energy consumption.

With the development of micro-processing technology, the functions of sensor nodes are becoming more and more powerful, and combined with Software Defined Network (SDN) technology, so that it has more vitality. At present, sensor-based networks have become an important basic network of the Internet of Things (IoTs). Due to the adoption of wireless technology, the spread of code in wireless sensor networks is a key issue for applications. It is important to automatically configure and upgrade software in industrial and environmental monitoring. However, how to quickly and efficiently spread code to the entire network is still a challenging issue. The fast transfer of program code becomes an important part of the solution. In this paper, we propose an AAS-LBR scheme that is far superior to previous strategies in the number of broadcasts. The AAS-LBR scheme has an important innovation that makes it far superior to previous strategies: it makes full use of the remaining energy of the far sink area to increase the active slot while expanding the broadcast radius. Because of the combination of these two methods, the AAS-LBR scheme of this paper has more flexibility in broadcasting, so that the number of broadcasts and transmission delay can be greatly reduced without affecting the network lifetime, which has been difficult to achieve in the past. 

## Figures and Tables

**Table 1:** Parameters of network.

Symbol	Description	Value
$\varepsilon_{s}$	Sleep power consumption	2.4 × 10^−7^ W
$S_{P}$	Preamble duration	0.26 ms
$S_{d}$	Packet duration	0.93 ms
$S_{al}$	Ack window duration	0.26 ms
$\varepsilon_{r}$	Reception power consumption	0.0588 W
γ	the probability of generating data	0.1

**Table 2:** The transmit range with output powers.

Rank (*k*)	Output Power (*q*(*k*))	Range (*r*(*k*))
0	−20 dBm (0.0100 mW)	28.0 m
1	−19 dBm (0.0126 mW)	29.7 m
2	−18 dBm (0.0158 mW)	31.4 m
3	−17 dBm (0.0200 mW)	33.3 m
4	−16 dBm (0.0251 mW)	35.3 m
5	−15 dBm (0.0316 mW)	37.4 m
6	−14 dBm (0.0398 mW)	39.6 m
7	−13 dBm (0.0501 mW)	41.9 m
8	−12 dBm (0.0631 mW)	44.4 m
9	−11 dBm (0.0794 mW)	47 m
10	−10 dBm (0.1000 mW)	49.8 m
11	−9 dBm (0.1259 mW)	52.8 m
12	−8 dBm (0.1585 mW)	55.9 m
13	−7 dBm (0.1995 mW)	59.2 m
14	−6 dBm (0.2512 mW)	62.7 m
15	−5 dBm (0.3162 mW)	66.4 m
16	−4 dBm (0.3981 mW)	70.3 m
17	−3 dBm (0.5012 mW)	74.5 m
18	−2 dBm (0.6310 mW)	78.9 m
19	−1 dBm(0.7943 mW)	83.6 m
20	0 dBm (1.0000 mW)	88.6 m
21	1 dBm (1.2589 mW)	93.8 m
22	2 dBm (1.5849 mW)	99.4 m
23	3 dBm (1.9953 mW)	105.2 m
24	4 dBm (2.5119 mW)	111.5 m
25	5 dBm (3.1623 mW)	118.1 m

**Table 3:** Notations.

$N_{i}\left(v\right)$	Set of nodes capable of communicating with node *v* at time slot i
R(v)	The root node of the subtree where node *v* is located
$U_{i}$	Set of nodes with active time-slot i
$C_{i}$	Set of covering nodes for $U_{i}$
P(v)	Set of nodes covering node *v*
C(v)	Set of nodes covered by node *v*

**Table 4:** Data packet dissemination using AAS-LBR.

Cycle	Slot	Data Received at Node
1	1	$v_{1}$
	2	$v_{2}$
2	0	$v_{15}$
	1	$v_{6}$, $v_{13}$
	2	$v_{3}$, $v_{14}$
3	0	$v_{5}$, $v_{7}$, $v_{10}$, $v_{16}$
	1	$v_{4}$, $v_{12}$, $v_{18}$, $v_{19}$
	2	$v_{8}$, $v_{9}$, $v_{21}$
4	0	$v_{11}$, $v_{17}$, $v_{20}$
	2	$v_{22}$

**Table 5:** Data packet dissemination using ABRCD.

Cycle	Slot	Data Received at Node
**1**	**1**	$v_{1}$
	2	$v_{2}$
2	0	$v_{15}$, $v_{6}$
	1	
	2	$v_{14}$
3	0	$v_{7}$, $v_{10}$, $v_{16}$
	1	$v_{12}$, $v_{13}$
	2	$v_{3},\;v_{8}$, $v_{9}$, $v_{19}$
4	0	$v_{4}$, $v_{5}$
	1	$v_{18}$, $v_{17}$
	2	$v_{19}$, $v_{21}$, $v_{22}$
5	0	$v_{20}$
	1	$v_{11}$

**Table 6:** Performance comparison.

The Scene or Parameter of Network	Scheme	The Number of Broadcast	The Reduce Ratio of Broadcast (%)	The Broadcast Delay	The Reduce Ratio of Delay (%)
R=500,|T| = 30, number of nodes are 200	AAS-LBR	87	29.83	37	40.32
	ABRCD	98	23.38	54	12.90
	LBAS	124	0	62	0
R=500,|T| = 90, number of nodes are 200	AAS-LBR	86	66.92	193	27.71
	ABRCD	189	27.30	243	8.98
	LBAS	260	0	267	0
R=500,|T| = 150, number of nodes are 200	AAS-LBR	89	68.55	420	32.69
	ABRCD	210	17.00	582	6.73
	LBAS	283	0	624	0
R=500,|T| = 20, number of nodes are 200	AAS-LBR	53	54.70	76	25.49
	ABRCD	81	30.80	89	12.74
	LBAS	117	0	102	0
R=500,|T| = 20, number of nodes are 400	AAS-LBR	78	37.10	92	29.77
	ABRCD	98	20.96	120	8.39
	LBAS	124	0	131	0
R=500,|T| = 20, number of nodes are 600	AAS-LBR	98	36.36	76	48.99
	ABRCD	143	6.54	136	8.72
	LBAS	154	0	149	0

**Table 7:** The energy consumption comparison.

The Scene or Parameter of Network	Scheme	The Maximum Energy Consumption (uj × $10^{3}$)	The Total Energy Consumption (uj × $10^{3}$)
R=500, |T| = 20, number of nodes are 200	AAS-LBR	155.97503	1295.02318
	ABRCD	155.97503	1036.77183
	LBAS	155.97503	1036.80625
R=500, |T| = 60, number of nodes are 200	AAS-LBR	409.47297	3397.00115
	ABRCD	409.47297	2727.33655
	LBAS	409.47297	2714.27026
R=500, |T| = 100, number of nodes are 200	AAS-LBR	662.6201	5504.79548
	ABRCD	662.6201	4415.58208
	LBAS	662.6201	4394.42601
